# First-in-Class Cyclic Temporin L Analogue: Design, Synthesis, and Antimicrobial Assessment

**DOI:** 10.1021/acs.jmedchem.1c01033

**Published:** 2021-07-23

**Authors:** Rosa Bellavita, Bruno Casciaro, Salvatore Di Maro, Diego Brancaccio, Alfonso Carotenuto, Annarita Falanga, Floriana Cappiello, Elisabetta Buommino, Stefania Galdiero, Ettore Novellino, Tom N. Grossmann, Maria Luisa Mangoni, Francesco Merlino, Paolo Grieco

**Affiliations:** † Department of Pharmacy, University of Naples “Federico II”, Naples 80131, Italy; ‡ Center for Life Nano- & Neuro-Science, Fondazione Istituto Italiano di Tecnologia (IIT), Rome 00161, Italy; § DiSTABiF, University of Campania “Luigi Vanvitelli”, Caserta 81100, Italy; ∥ Department of Agricultural Sciences, University of Naples “Federico II”, Portici 80055, Italy; ⊥ Department of Biochemical Sciences, Laboratory affiliated to Istituto Pasteur Italia-Fondazione Cenci Bolognetti, Sapienza University of Rome, Rome 00185, Italy; # Department of Chemistry & Pharmaceutical Sciences, VU University Amsterdam, Amsterdam 1081 HZ, The Netherlands

## Abstract

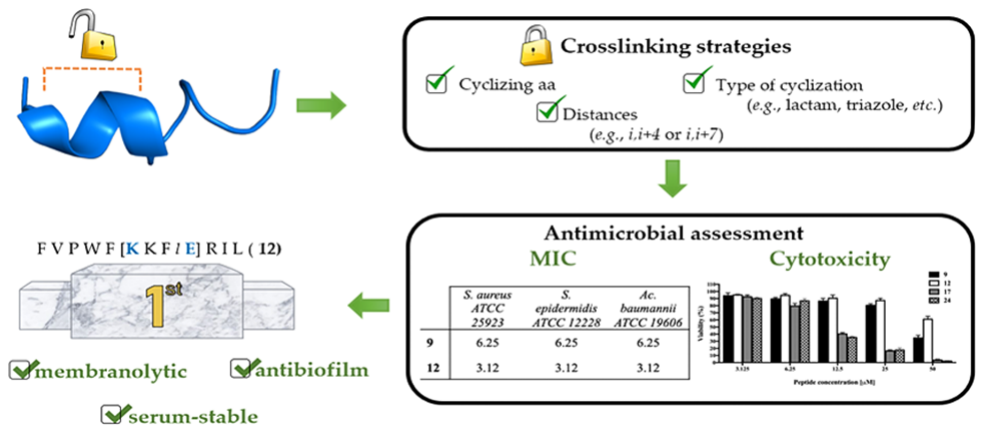

The pharmacodynamic and pharmacokinetic properties of bioactive peptides can be modulated by introducing conformational constraints such as intramolecular macrocyclizations, which can involve either the backbone and/or side chains. Herein, we aimed at increasing the α-helicity content of temporin L, an isoform of an intriguing class of linear antimicrobial peptides (AMPs), endowed with a wide antimicrobial spectrum, by the employment of diverse side-chain tethering strategies, including lactam, 1,4-substituted [1,2,3]-triazole, hydrocarbon, and disulfide linkers. Our approach resulted in a library of cyclic temporin L analogues that were biologically assessed for their antimicrobial, cytotoxic, and antibiofilm activities, leading to the development of the first-in-class cyclic peptide related to this AMP family. Our results allowed us to expand the knowledge regarding the relationship between the α-helical character of temporin derivatives and their biological activity, paving the way for the development of improved antibiotic cyclic AMP analogues.

## Introduction

Peptide macrocyclization is a well-established strategy for tuning pharmacodynamic and pharmacokinetic (PK) properties of peptide-based molecules of therapeutic interest.^1^ Among the most popular cyclizations, head-to-tail, e.g., yielding homodetic peptides, and side-chain-to-side-chain tethering grafted by lactam, disulfide, and 1,4 or 1,5-triazolic bridges, have been successfully employed to reduce conformational flexibility of biological active linear sequences, hence improving the binding affinity and specificity to targets, as well as membrane interaction and cell permeability.^1−7^ To date, many cyclic natural and synthetic peptides are under investigation for therapeutic purposes,^8^ such as anticancer,^9^ antifungal,^10^ antiviral,^11^ anti-inflammatory,^12^ and antimicrobial one.^13^ As a consequence, the number of approved peptide-based drugs is progressively increasing.^14^ For instance, romidepsin (i.e., a 5-mer cyclic depsipeptide), Istodax, a well-known HDAC inhibitor acting as a potent anticancer drug, is used in T-cell lymphoma;^15^ micafungin (i.e., a cyclic hexapeptide), Mycamine, approved as antifungal agent, is effective against *Candida* infections;^16^ and alisporivir (homodetic cyclic undecapeptide), a cyclophilin inhibitor used in the treatment of hepatitis C (HCV), has passed the phase II clinical trials as antiviral drug.^17^

**Chart 1:**
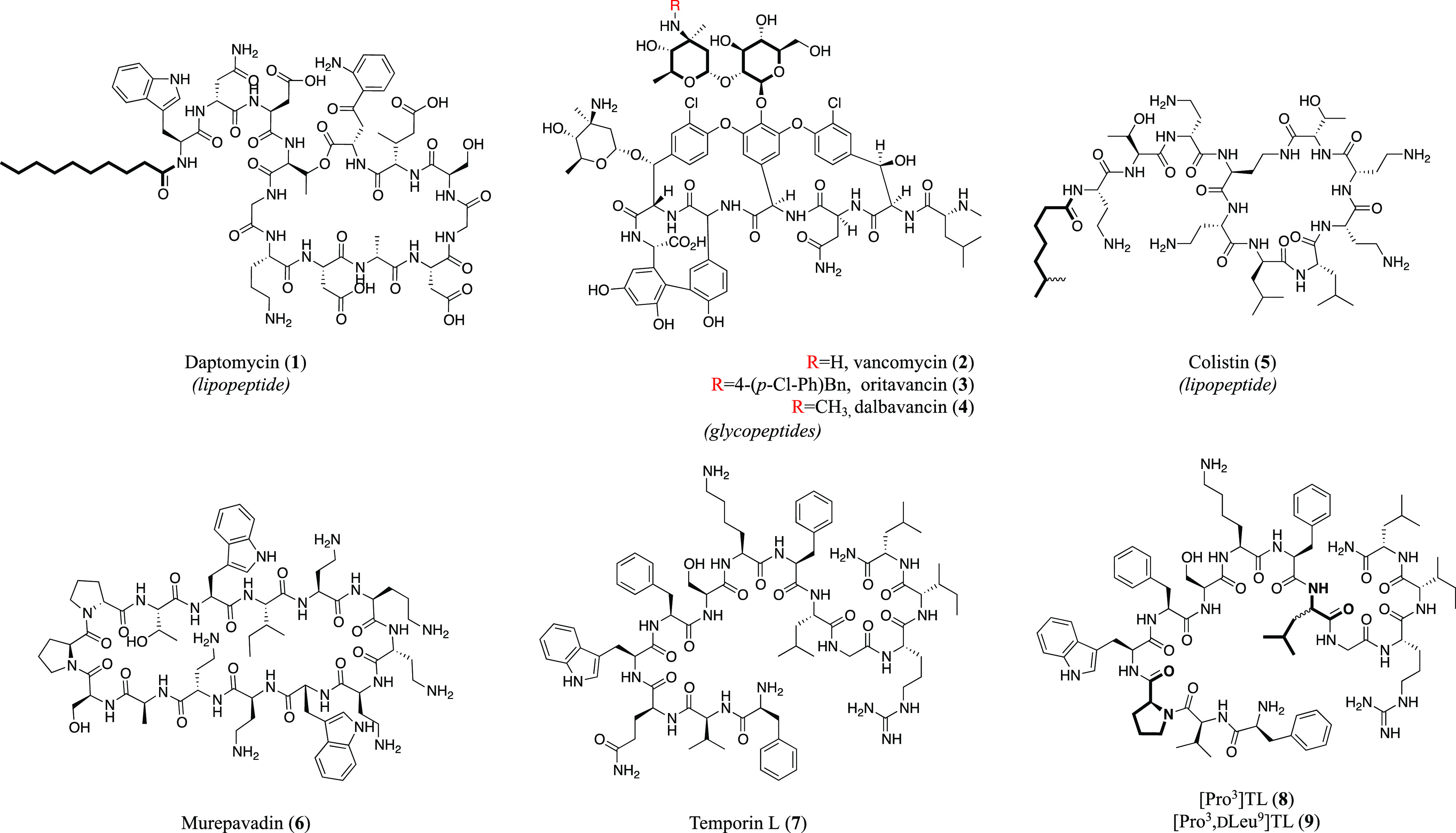
Examples of Macrocyclic Peptide-Based Antibiotic Molecules (**1**–**9**), such as Lipopeptides (**1** and **5**), Glycopeptides (**2**–**4**), and Others, Including Temporin L (**7**) and Its Derivatives [Pro^3^]TL (**8**) and [Pro^3^,dLeu^9^]TL (**9**)a

Noteworthy, in the field of antimicrobial peptides (AMPs) drug discovery,^18^ the use of macrocyclic peptide-based molecules represents an attractive strategy,^19^ considering both their different mechanism of action compared to the available antibiotics and their improved drug-like features with respect to the linear antimicrobial counterparts (e.g., proteolytic stability). Nevertheless, a restricted number of cyclic peptides have been approved for therapy, including natural and synthetic cyclic AMPs. Prominent examples are daptomycin (**1**),^20^ a cyclic lipopeptide, and vancomycin (**2**),^21^ together with its derivatives oritavancin (**3**) and dalbavancin (**4**) (Chart 1),^22^ as cyclic glycopeptides, which are all used to treat infections caused by Gram-positive bacteria, including multidrug-resistant strains. Others, albeit a few, are described as anti-Gram-negative bacteria, such as colistin (**5**),^23^ a polymyxin lipopeptide produced by *Aerobacillus colistinus* that was approved by the Food and Drug Administration (FDA) and characterized by a potent and specific activity against several Gram-negative bacteria, including *Pseudomonas aeruginosa* and *Acinetobacter baumannii*; and murepavadin (**6**), a synthetic cyclic β-hairpin peptidomimetic identified as *Pseudomonas*-specific antibiotic (Chart 1).^24^

a **9** was subjected to a macrocyclization design during our study.

AMPs are active also against fungi and viruses and may act through several mechanisms of action, e.g., via membrane disruption and/or intracellular targets.^25^ Their cyclic derivatives can confer unique conformational features addressing the main peptide limitations, including poor oral bioavailability and proteolytic stability.^26^ Therefore, the synergy between this emerging class of therapeutics with the most advanced chemical manipulations could drive toward novel therapeutic approaches and discovery of molecules useful in the fight against infectious diseases.^27^ In addition, it is now well known that if no prompt action is taken against some “critical” pathogens such as the carbapenem-resistant *A. baumannii*, *P. aeruginosa*, and methicillin-resistant *Staphylococcus aureus*, and related antibiotic resistance phenomenon, about 10 million deaths per year could be caused over the next 30 years, as reported by the World Health Organization (WHO).^28^

Temporins are AMPs and belong to the group of host defense peptides (HDP), originally isolated from the amphibian skin secretions of the red frog *Rana temporaria*.^29^ Their sequences are composed of 10–14 amino acids, amidated at the C-terminus. In hydrophobic environments, temporins fold into an amphipathic α-helix that, along with their cationic nature at physiological pH (from +2 to +3), is accountable for their biological behavior. Specifically, temporins are membrane-misfolding agents, hence bypassing the opportunity for the bacteria to acquire resistance, which act against Gram-positive strains with a minimal inhibitory concentration (MIC) ranging from 2.5 to 20 μM. Temporin L (TL, **7**) (Chart 1), FVQWFSKFLGRIL, is considered the most promising candidate among the ∼130 isoforms of this family due to its potent activity against microorganisms including Gram-negative bacteria, such as *P. aeruginosa* and *Escherichia coli*.^29,30^ Functional investigations demonstrated that **7** was able to interfere with *E. coli* divisome machinery, binding to the FtsZ protein (*K*_D_ = 17.4 ± 0.8 nM) and inhibiting its GTPase activity.^31^ TL has proven to mediate a strong antibiofilm activity against *P. aeruginosa* PAO1 and methicillin-resistant *S. aureus*,^32,33^ but the underlying molecular mechanism is still unknown. The resulting effect might be due to either interference with biofilm formation or downregulation of genes involved in the synthesis of biofilm components.^34^ Furthermore, TL has exhibited synergistic anticancer, antiendotoxin, and immunomodulatory activities, when tested with other temporin members (e.g., isoforms A and B)^35^ but has also displayed a significant hemolytic activity at microbicidal concentrations.^36^ For this reason, TL has been extensively studied as a model peptide to develop several derivatives with improved bioactivity and limited toxicity.^37−39^ Many local modifications upon its sequence were performed to ameliorate its therapeutic index, by intervening on the membrane interaction [e.g., promoting the self-assembling in lipopolysaccharides (LPS) and reducing the aggregation state in aqueous environment]^40^ or on the secondary structure (e.g., modulating the nonmembrane-lytic or membrane-lytic mechanism of action).^41^ Indeed, from a conformational point of view, the α-helical secondary structure along the entire sequence, particularly at the *N*-terminus, is known to be responsible for its activity, including toxicity, as demonstrated by structural studies focused on the interaction between TL and both negatively charged sodium dodecyl sulfate (SDS) and zwitterionic dodecylphosphocholine (DPC) micelles, mimicking bacterial and mammalian membranes, respectively.^42−44^ However, during previous structure–activity relationship (SAR) campaigns, the replacement of Gln^3^ with Pro, [Pro^3^]TL (**8**),^42^ and the simultaneous stereoinversion of Leu^9^, [Pro^3^,dLeu^9^]TL (**9**),^45^ generated analogues that featured a reduction of hemolysis and a retained antimicrobial activity, especially against Gram-positive bacteria. Interestingly, such modifications, both taken as α-helical breakers, provoked distinctive effects as the only impairment of the *N*-terminus framework by the Pro^3^ incorporation did not significantly affect the antimicrobial spectrum but slightly reduced the toxicity; while the simultaneous disruption of the α-helical motif in position 9 by the incorporation of d-Leu conferred a dramatic loss of hemolysis, albeit a coinciding weaker microbicidal performance. In this scenario, the adjustment of the α-helical content in the C-terminus represents a crucial element for determining the best balance between antimicrobial activity and toxicity toward human erythrocytes and keratinocytes.

In the light of these considerations, we hypothesized that the α-helix stabilization in the C-terminus region of **9**, by means of an intramolecular macrocyclization strategy, could lead to novel derivatives with potentially increased antimicrobial spectrum and lower cytotoxicity.

Notably, one of the most straightforward stapling strategies for the helix stabilization consists of a side-chain-to-side-chain tethering by the incorporation of precursor amino acids in i,i+4 or i,i+7 positions, to bridge one or two helical turns, respectively.^5,6,46−48^ For that reason, we herein applied chemical tethering between side chains belonging to diverse residues placed in different positions along the linear sequence of **9**. After identification of suitable linking sites, a variety of side-chain tethering strategies were embraced to further evaluate the correlation between α-helical content and biological activity. The entire peptide library was assessed by antimicrobial assays, allowing us to select certain derivatives, which were tested for cytotoxicity and analyzed by circular dichroism (CD). Based on these results, fluorescence-based studies (Thioflavin T and Laurdan assays) were performed to shed light on the mechanism of action of the first-in-class active cyclic TL analogue, peptide **12**, which was further assessed by membrane leakage and time kill assays, as well as for its antibiofilm activity against relevant human pathogens. Moreover, **12** underwent additional conformational analyses by nuclear magnetic resonance (NMR) spectroscopy and, finally, the PK impact of the stapling strategy was probed by a protease stability experiment carried out in the presence of human serum.

## Results

### Design Strategy

**Figure 1:**
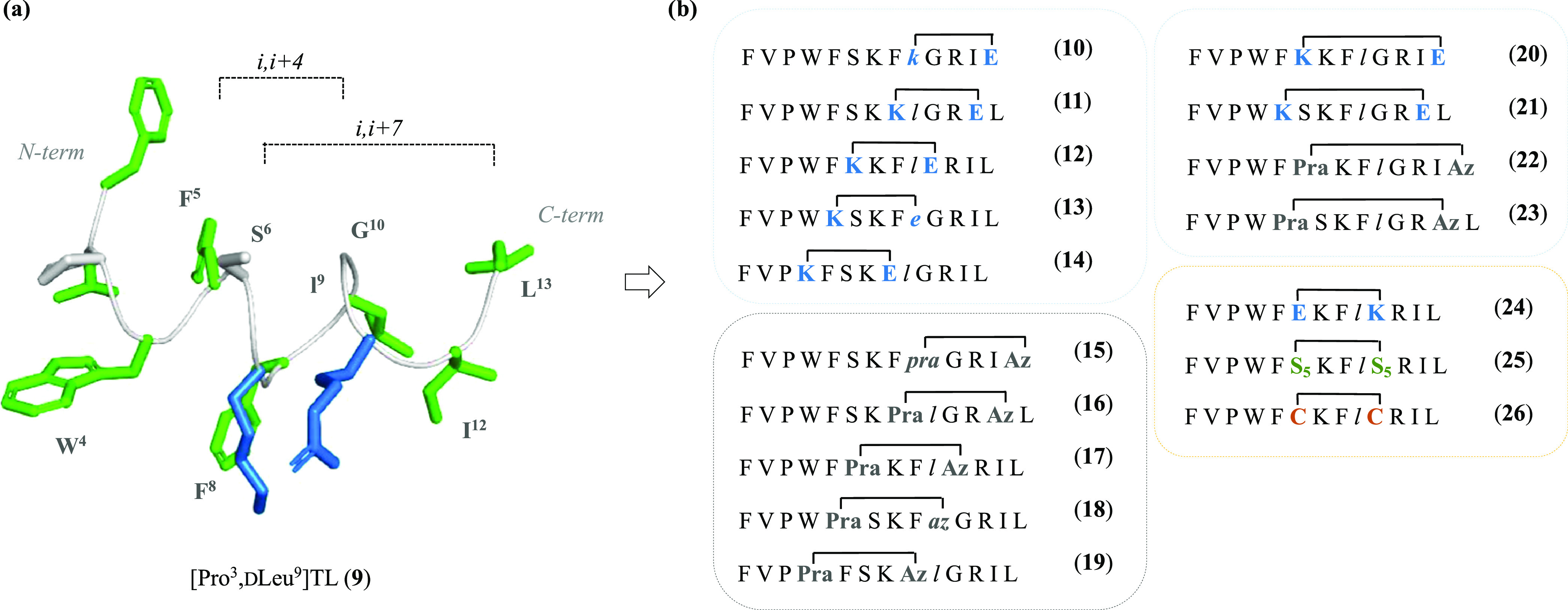
(a) Three-dimensional (3D) structure of peptide **9** from previous NMR analysis taken in consideration for the design of cyclic peptide derivatives (PDB ID 7OS8). Peptide **9** was depicted as sticks and ribbons (green, hydrophobic residues; blue, basic residues; gray, backbone). (b) Sequences of the designed peptides **10**–**26** featuring diverse types of cyclization and distances. Lowercase letters in italic mean d isomers. Pra = propargylalanine; Az = azidolysine; S_5_ = (*S*)-2-(4-pentenyl)alanine.

The design of our cyclic temporin derivatives was inspired by the crucial role of the α-helix content in the C-terminus of temporin L and analogues, as described in previous works.^45^ Thus, peptide **9** was selected as model sequence, and its C-terminal region underwent several modifications to introduce amino acid residues suitable for side-chain-to-side-chain tethering (Figure 1). As proof of concept for chemical stabilization of the α-helical structure, we first reasoned upon two systematic modifications via the coupling-based lactamization and the azide–alkyne cycloaddition. These reactions were performed on solid-phase employing allyl-protected lysine and glutamic acid [Lys(Alloc)/Glu(OAll)] or ω-alkynyl and azidolysine [propargylalanine (Pra)/Lys(N_3_)] pairs, to create lactam and 1,4-triazolic bridges, respectively.^2,49^ The incorporation of lactam and triazolic linkers was also expected to impact on the physicochemical (polarity contribution) and PK properties (protease resistance).^50^ The design then proceeded according to the following modification steps: (i) identification of the residues to replace to allow side-chain tethering in i,i+4 positions; (ii) definition of the right distance between cyclizing amino acids, i.e., i,i+4 and i,i+7, corresponding to one or two helical turns, respectively; and (iii) application of different types of cyclization. Considering previous conformational NMR studies performed in both SDS and DPC,^42−44^ the linear [Pro^3^,dLeu^9^]TL (**9**) peptide displayed an α-helical structure along residues 5–8 in SDS and residues 5–10 in DPC; while in both micelles, it showed a β-turn centered on Pro^3^ and Trp^4^.^41^ In light of these results, we started replacing Trp^4^ with Lys (i) or Pra (i), and Phe^8^ with Glu (i+4) or Lys(N_3_) (i+4), until C-terminal residues, yielding the first series of lactam (**10–14**) and 1,4-triazolic (**15**–**19**) bridged derivatives. During the systematic incorporation of cyclizing amino acids, positively charged Lys^7^ and Arg^11^ residues were not taken in consideration because of their essential role for membrane interaction. Also, a d-residue in position 9 was still considered for cyclizing amino acids replacing the original d-Leu. To explore additional secondary structure variations, the lactam and triazole stapling at i,i+7 positions were also obtained (compounds **20**–**23**). Finally, once positions Ser^6^ and Gly^10^ were identified as suitable for cyclization, they were further investigated by considering the inversion of positions of Lys and Glu amino acids (**24**), and the hydrocarbon (**25**) and disulfide (**26**) linkers.^51,52^

### Synthesis

**Scheme 1:**
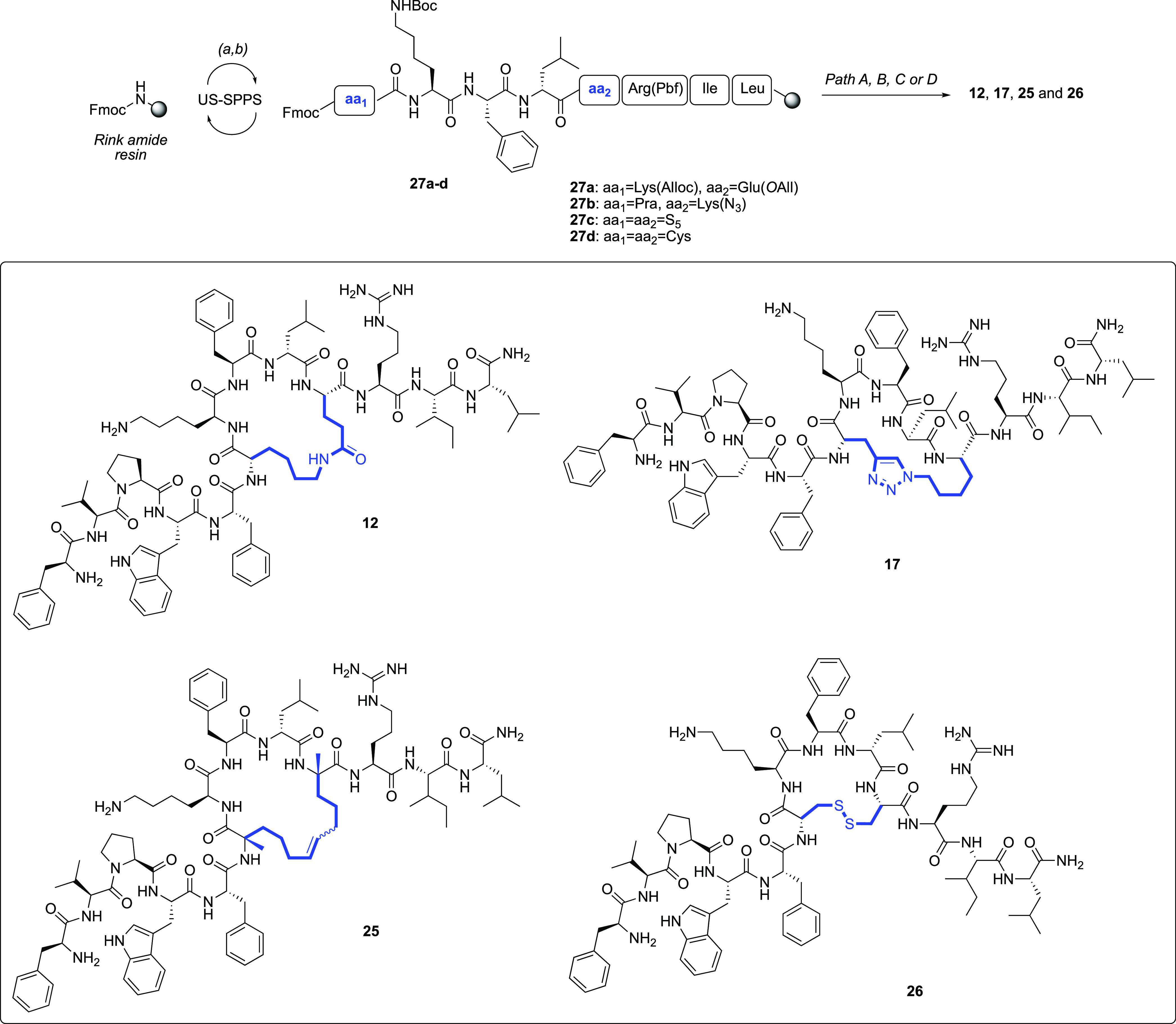
Synthetic Strategy for Achieving Representative Compounds **12**, **17**, **25**, and **26** (a) US–SPPS—Fmoc deprotection: 20% piperidine in DMF, 0.5 + 1 min; (b) US–SPPS—coupling: Fmoc-AA, COMU, Oxyma, DIEA, 5 min. See Scheme S14, for paths A (lactamization), B (CuAAC reaction), C (RCM reaction), and D (disulfide formation), embraced for the syntheses of **12**, **17**, **25**, and **26**, respectively, as well as for the entire peptide library.

Linear precursors of peptides **10**–**26** were synthesized following the Fmoc-based ultrasonic-assisted solid-phase peptide synthesis (US–SPPS) methodology.^53^ The US–SPPS was employed for the Fmoc deprotection (20% piperidine in dimethylformamide (DMF), 0.5 + 1 min) and the coupling (COMU/Oxyma as activating/additive agents, 5 min treatment) reactions, which were cyclically performed until the accomplishment of the resin-bound target linear peptide sequences (Scheme 1, see paths A–D in Scheme S14).

#### Lactam-Stapled Peptides (**10**–**14**, **20**, **21**, and **24**)

The lysine and glutamic acid side-chain allylic protection groups were selectively removed as previously described.^54^ In particular, the palladium-catalyzed reaction was carried out on solid-phase intermediates and subsequent intramolecular amide bond formation was achieved using (7-azabenzotriazol-1-yloxy)tripyrrolidinophosphonium hexafluorophosphate (PyAOP) and 1-hydroxyazabenzotriazole (HOAt).^55^

#### 1,4-Disubstituted [1,2,3]-triazolic Peptides (**15**–**19**, **22**, and **23**)

The 1,4-disubstituted triazolyl bridge was obtained via an intermolecular copper-catalyzed azide–alkyne cycloaddition (CuAAC) reaction,^49,56^ between the azidolysine [Lys(N_3_)] and the *N*-terminal Fmoc-protected Pra residues of shorter intermediates, such as **27b**. Then, the sequence was further elongated according to the procedures mentioned above.^53^

#### Hydrocarbon-Stapled Peptide (**25**)

Tethering by alkene bridge in peptide **25** was attained by performing the on-resin ring-closing olefin metathesis (RCM) reaction.^57^ The resin was treated with a solution of the Grubbs’ first-generation catalyst in 1,2-dichloroethane (DCE), under constant bubbling nitrogen. The formation of olefinic bridge was monitored by liquid chromatography–mass spectrometry (LC–MS) analysis.

#### Disulfide-Bridged Peptide (**26**)

Peptide **26** was cleaved from the resin as linear sequence upon treatment with a solution of trifluoroacetic acid (TFA) and triisopropylsilane (TIS). The oxidation of sulfide side chains was achieved by dissolving the crude peptide in H_2_O, considering a final peptide concentration of 0.5 mM, and then an aqueous solution of *N*-chlorosuccinimide (NCS) was added.^58^

After cleavage, crude peptides **10–26** were purified by reversed-phase high-pressure liquid chromatography (RP-HPLC). Each peptide (**10**–**26)** was assessed for purity >97% by analytical RP-HPLC, and the correct molecular mass was confirmed through high-resolution mass (HRMS) spectrometry (see Table S1).

### Minimal Inhibitory Concentration (MIC)

The antimicrobial activity of peptides **9**–**26** was assessed against a range of reference bacterial strains such as *S. aureus* ATCC 25923, *Staphylococcus epidermidis* ATCC 12228, *Bacillus megaterium* Bm11, *E. coli* ATCC 25922, *P. aeruginosa* ATCC 27853, and *A. baumannii* ATCC 19606 by the broth microdilution assay to determine the minimal growth inhibitory concentration (MIC, Table 1).^59^ First, we evaluated the effects produced by the lactam bridge between Lys and Glu residues placed at different positions along the peptide sequence of reference **9**. As indicated in Table 1, peptides **10** and **11** were not effective against the Gram-negative strains (MIC > 100 μM), while weakly worked against the Gram-positive strain of *B. megaterium* Bm11 (MIC, 50 μM). Conversely, peptide **12** resulted to be potent toward *B. megaterium* Bm11 (MIC, 1.56 μM) and even more active than reference peptide **9** against *S. aureus* ATCC 25923 and *S. epidermidis* ATCC 12228 (MIC, 3.12 μM), emerging as the most promising cyclic AMP of the lactam bridge series. In fact, the remaining lactam derivatives, **13** and **14**, did not show any antimicrobial effect up to 100 μM on all of the tested bacterial strains. Next, the 1,4-triazolyl series (**15**–**19**) generally displayed a significant reduction of antimicrobial activity (MIC > 100 μM), with the sole exception of peptide **17**, which was more active against Gram-positive strains *S. aureus* ATCC 25923 and *S. epidermidis* ATCC 12228 (MIC, 3.12 μM) than the parent peptide **9**. When the i,i+7 cyclization strategy was applied (**20**–**23**), a dramatic decrease of bacterial inhibition activity was observed. Specifically, the peptides **20**, **22**, and **23** only affected the microbial growth of the Gram-positive *B. megaterium* Bm11, at 12.5 μM. Among the peptides from the last-stage design strategy, **24**, endowed with a reverse orientation of lactam bridge with respect to peptide **12**, conserved an antimicrobial activity comparable to its parent peptide, being effective toward *S. aureus* ATCC 25923 (MIC, 3.12 μM) and *S. epidermidis* ATCC 12228 (MIC, 1.56 μM) and toward the Gram-negative *A. baumannii* (MIC, 3.12 μM). Peptide **25**, which differs from **9** by the presence of an olefin bridge rather than lactam one, dramatically lost the activity (MIC > 100 μM) vs both Gram-positive and Gram-negative strains. In contrast, peptide **26** preserved the activity of the parent peptide **9** toward *S. epidermidis* ATCC 12228 (MIC, 6.25 μM) and *B. megaterium* Bm11 (MIC, 1.56 μM), while it resulted to be inactive against *S. aureus* ATCC 25923 (MIC, 100 μM). As for the Gram-negative strains, this peptide exhibited a weak activity toward *E. coli* ATCC 25922 and *A. baumannii* ATCC 19606 (MIC, 25 μM) and did not affect *P. aeruginosa* ATCC 27853 growth (MIC > 100 μM).

**Table 1:**
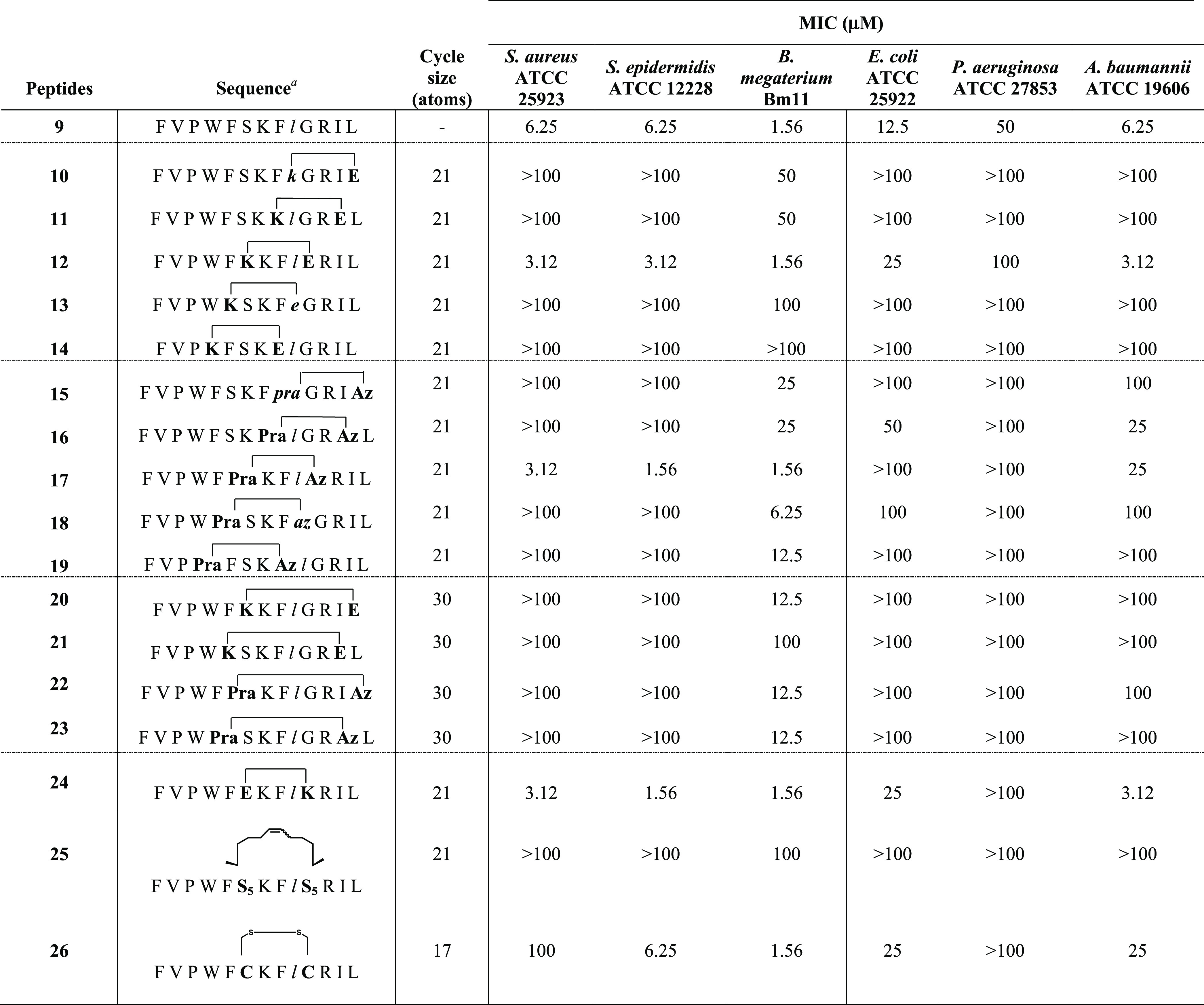
Antimicrobial Activity of the Designed Cyclic **9**-Derived Peptides^b^

a Lowercase letters in italic indicate amino acids in d-configuration. Pra = propargylalanine; Az = azidolysine; S_5_ = (*S*)-2-(4-pentenyl)alanine.

b Aliquots (50 μL) of bacteria in mid-log phase (final concentration of 1 × 10^6^ colony-forming units (CFU)/mL) in the Mueller–Hinton broth (MH) were added to 50 μL of MH broth containing the peptide in serial twofold dilutions. The minimal inhibitory concentration (MIC) was defined as the concentration (μM) of peptide at which 100% inhibition of microbial growth was observed, after an incubation of 16–18 h at 37 °C.

### Cytotoxicity

**Figure 2:**
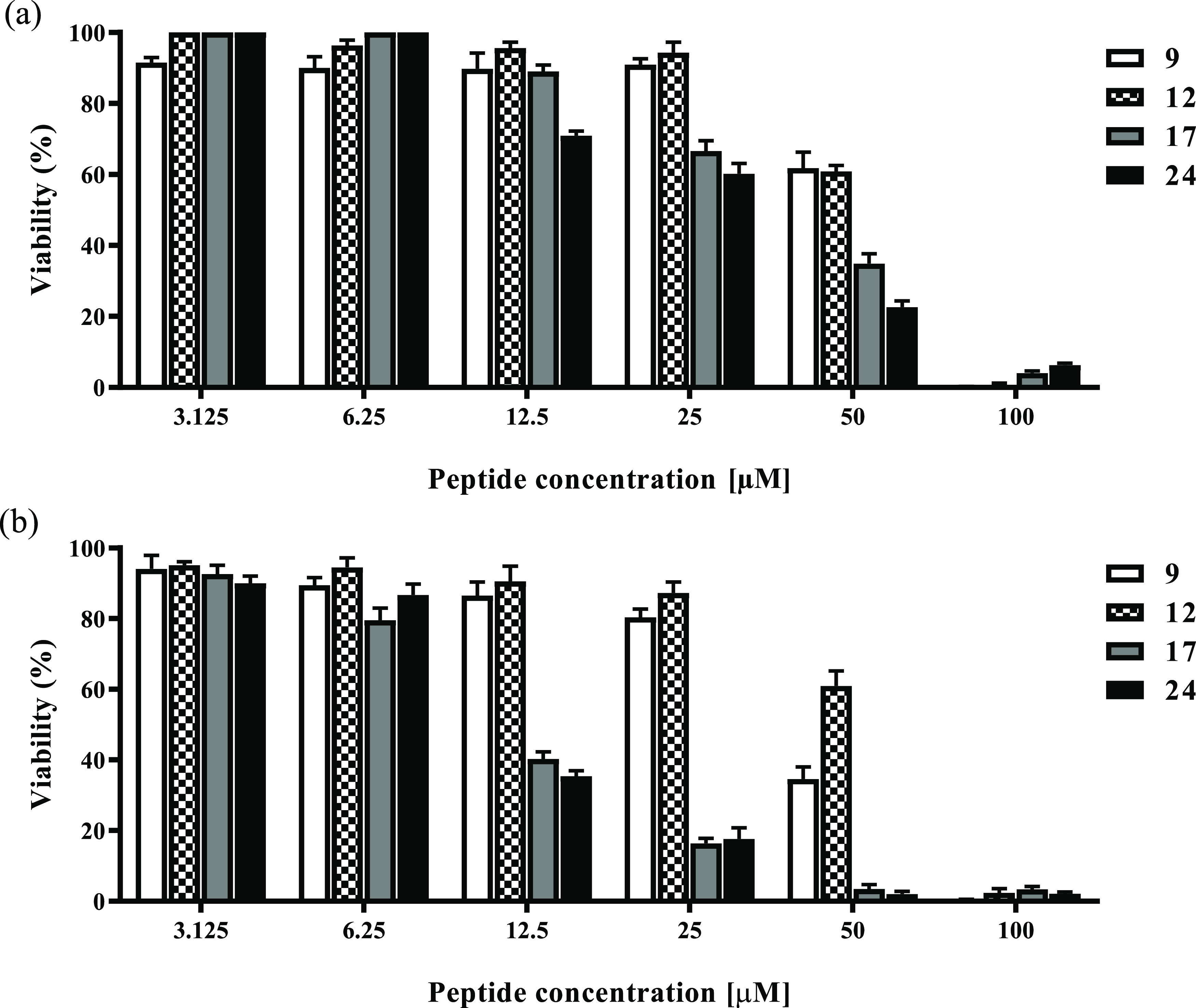
Viability of peptide-treated HaCaT cells evaluated by MTT assay at 2 h (a) and 24 h (b). All data are expressed as a percentage with respect to the untreated control cells and are the mean of three independent experiments ± standard error of the mean (SEM).

The extent of cytotoxic effects of antimicrobial peptides **9**, **12**, **17**, and **24** was assessed on keratinocytes (HaCaT cells), the prevalent cell types of human epidermis and that can be infected by microbes including both Gram-positive and Gram-negative bacteria. In particular, the cytotoxicity was evaluated by the 3-(4,5-dimethylthiazol-2-yl)-2,5-diphenyltetrazolium bromide (MTT) assay and is expressed as the percentage of viability of peptide-treated cells compared to that of untreated control cells. As expected, after 2 and 24 h treatments, the cell viability decreased in a concentration-dependent manner for all peptides (Figure 2). Among these, peptides **17** and **24** presented the strongest cytotoxicity as their cell viability after 2 h (Figure 2a) resulted in the range of 60–70% at the 25 μM concentration, less than 40% at the 50 μM concentration, and even less than 20% after 24 h treatment (Figure 2b). Interestingly, peptide **12** showed the same cytotoxic profile after either 2 or 24 h treatments at all concentrations tested. In particular, peptide **12** led to the highest cell viability (94% at 25 μM and 60% at 50 μM), highlighting the best safety profile, also compared to the reference compound **9**.

### Circular Dichroism (CD)

The secondary structures of the linear peptide **9** and its stapled analogues **12**, **17**, and **24–26** were explored by circular dichroism (CD). CD spectroscopy studies were performed in water, and SDS or DPC micelles (20 mM) were used as mimics of the bacterial or mammalian membranes, respectively.^60^

**Figure 3:**
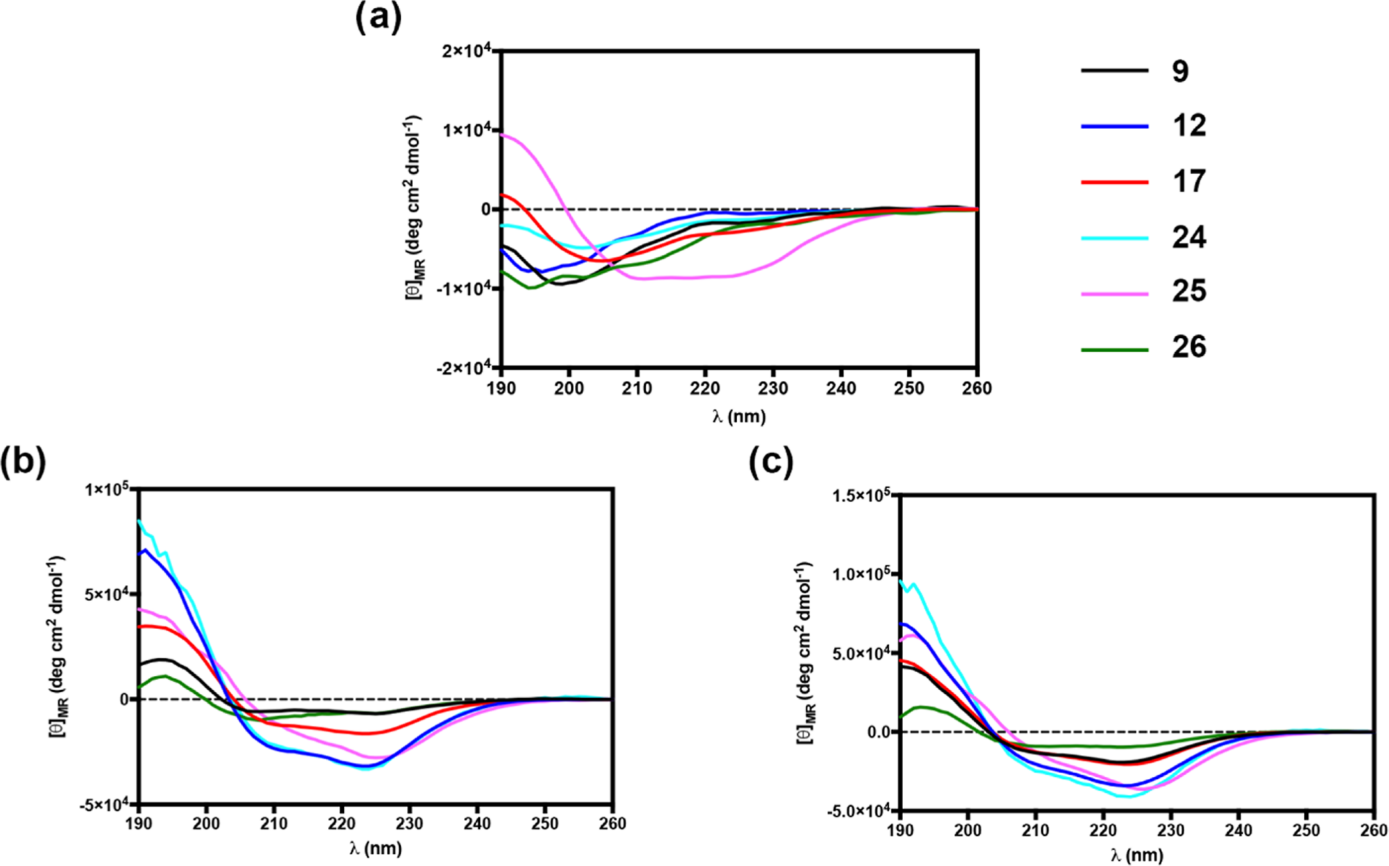
(a) CD spectra of peptide **9** and its cyclic analogues **12**, **17**, **24**, **25**, and **26** in water. (b, c) CD spectra of peptides **9**, **12**, **17**, **24**, **25**, and **26** in SDS and in DPC micelles, respectively.

The secondary structure content was predicted based on the CD spectra from 190 to 260 nm using the online server for protein secondary structure analyses, DichroWeb.^61^ CD spectra in water revealed the prevalence of disordered conformers for compounds **9**, **12**, **17**, **24**, and **26** with a minimum close to 200 nm (Figure 3a). The hydrocarbon-bridged peptide **25** was slightly helical and structured in water, with weak ellipticity at 220 nm. Peptide **25** contains ∼13% regular helix and ∼10% distorted helix, according to the DichroWeb prediction. However, noncanonical dichroic shape of the CD spectrum strongly suggests a tendency of **25** to form α-helical aggregates in water solution. CD spectra in SDS micelles showed two minima close to 208 and 222 nm, indicating a higher helical propensity, except for the disulfide-bridged peptide **26** (Figure 3b). CD spectra of peptide **26** and its linear precursor **9** showed the lowest helical percentage according to the DichroWeb prediction (Table 2). This result revealed that the disulfide bridge did not induce a significant helicity in i,i+4 positions as there is no difference in helical content with its linear precursor **9**. In the presence of SDS, the lactam-linked peptides **12** and **24** showed very similar CD spectra and together with the hydrocarbon-stapled peptide **25** showed the highest content of regular (∼58 and ∼41% for **12**/**24** and **25**, respectively) and distorted (∼24 and ∼22% for peptides **12/24** and **25**, respectively) α-helix, followed by 1,4-triazolic-bridged peptide **17** with ∼32% regular and ∼16% distorted α-helix according to the DichroWeb prediction (Table 2).

**Table 2:** Secondary Structure^a^ Percentages of **9** and Its Cyclic Analogues in Water, SDS, and DPC Micelles

	water						SDS solution						DPC solution					
peptides	9	12	17	24	25	26	9	12	17	24	25	26	9	12	17	24	25	26
helix-1	0	0	2	1	13	1	16	58	32	59	41	14	36	54	38	69	54	18
helix-2	3	2	4	6	10	7	12	24	16	18	22	11	17	21	18	23	34	14
β-strand-1	26	18	19	16	12	13	14	0	13	11	0	12	9	1	9	0	0	9
β-strand-2	11	8	9	9	8	9	8	3	7	5	4	8	7	4	7	3	7	7
β-turn	21	15	16	17	16	19	15	10	15	7	5	17	12	7	10	4	5	17
random	38	55	49	50	41	50	34	5	16	0	28	37	18	13	19	0	0	35

a Helix 1, helix 2, strand 1, and strand 2 indicate a regular α-helix, distorted α-helix, regular β-strand, and distorted β-strand, respectively.

In DPC micelles, all peptides revealed two minima close to 208 and 222 nm, indicating helical propensity (Figure 3c). CD spectra showed that disulfide-bridged peptide **26** was less helical in DPC with weak ellipticity at 220 nm and the lowest percentage of helical content according to the DichroWeb prediction (Table 2). As reported in Table 2, lactam-stapled peptides **12** and **24**, and hydrocarbon-stapled peptide **25** displayed the highest content of regular (69–54%) and distorted (21–34%) α-helix, followed by triazole-bridged peptide **17** showing a content of ∼38% regular and ∼18% distorted α-helix.

### Interactions between Cell Membrane and Peptides **9**, **12**, and **17**

**Figure 4:**
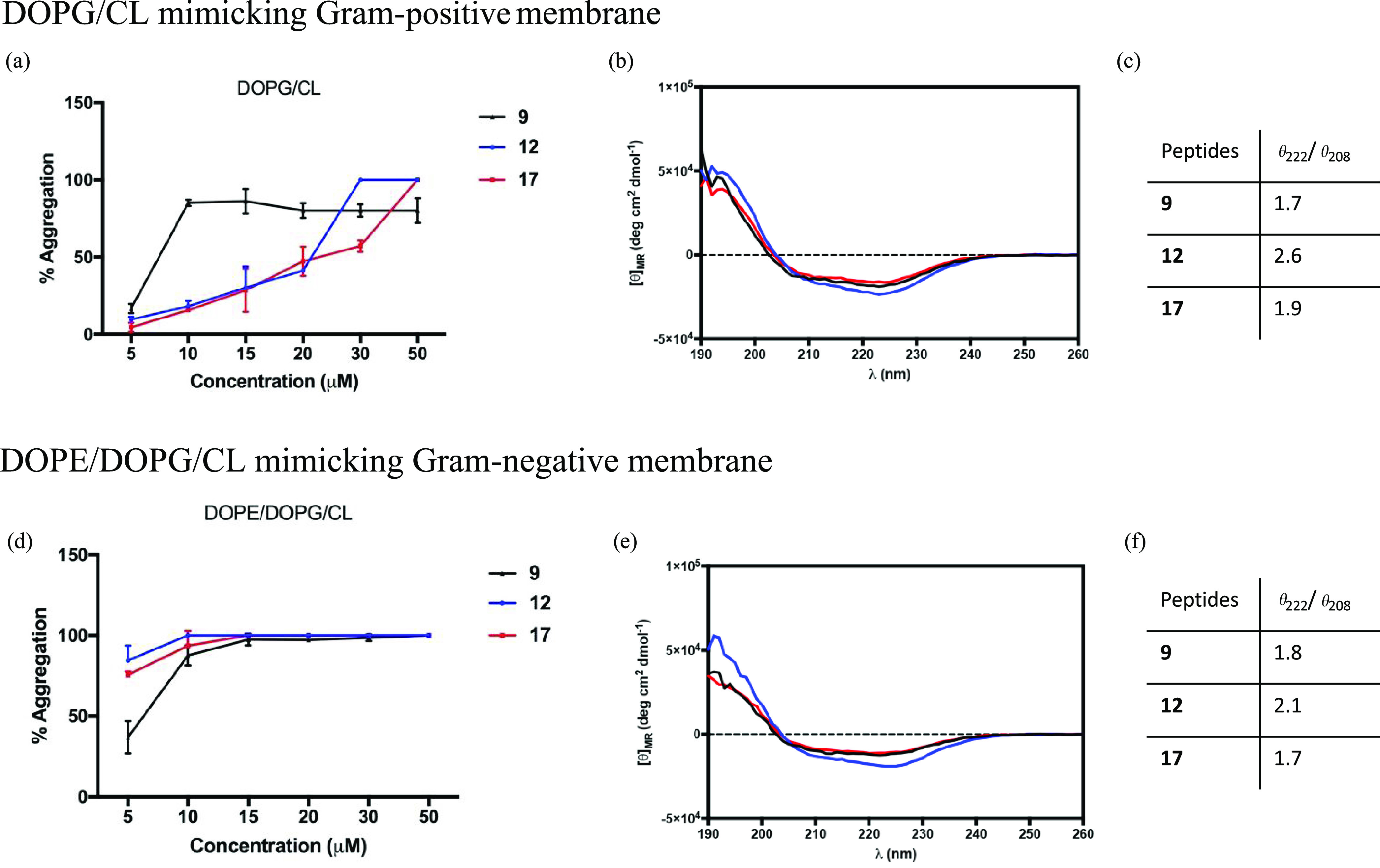
(a, d) Percentage of aggregation as a function of peptide concentration by monitoring ThT emission and CD spectra of peptides **9**, **12**, and **17** in the presence of liposomes mimicking Gram-positive (a–c) and Gram-negative (d–f) membranes. (b, e), CD spectra of peptides **9**, **12**, and **17** in SUVs (**9**, black line; **12**, blue line; **17**, red line). (c, f) Table reports the ratio of the ellipticities at 222 and 208 nm, which discriminates between monomeric and oligomeric states of helices for peptides **9**, **12**, and **17**.

Different antimicrobial behavior of cyclic peptides **12** and **17** was probed by evaluating their interaction mode with bacterial membrane, in comparison with the linear peptide **9**. The Thioflavin T (ThT) fluorescent probe was used to measure the tendency of peptides **9**, **12**, and **17** to oligomerize in the presence of the bacterial membrane because it is highly sensitive to molecular aggregation phenomena resulting in an enhancement of fluorescence intensity.^62,63^ We used large unilamellar vesicles (LUVs) made of phosphatidylethanolamine (DOPE), phosphatidylglycerol (DOPG), and cardiolipin (CL) (63/23/12) to mimic lipid composition of the cytoplasmic membrane of Gram-negative bacteria. Gram-positive membranes were otherwise mimicked using LUVs made of DOPG and CL (58/42). A significant increase of ThT fluorescence was observed for LUVs mimicking Gram-positive bacteria after the addition of linear peptide **9** at a concentration of 10 μM (Figure 4a–c). In contrast, the lactam-bridged peptide **12** and triazole-bridged peptide **17** exhibited a progressive phenomenon of aggregation in DOPG/CL LUVs, in particular, at 30 and 50 μM, respectively. Remarkably, in liposomes mimicking Gram-negative membranes, all peptides showed the same trend (Figure 4d–f), and we observed an intense increase of ThT fluorescence emission at all peptide concentrations reaching the plateau after the addition of 10 μM of all analyzed peptides.

The oligomeric states of peptides **9**, **12**, and **17** were also characterized by CD spectroscopy in the presence of liposomes mimicking Gram-positive and Gram-negative membranes (Figure 4b,e). All peptides had a high propensity to give helical aggregates in both Gram-positive and Gram-negative membranes, as indicated by two negative bands at about 208 and 222 nm. The ratio of the ellipticities at 222 and 208 nm was calculated to discriminate between monomeric and oligomeric states of helices.^64^ As observed in our CD spectra, the ratio θ_222_/θ_208_ is always greater than 1.0 in both membranes, indicating an α-helical conformation in its oligomeric state (Figure 4c,f).

### Aggregation Mode in the Interaction between Peptides and LPS

**Figure 5:**
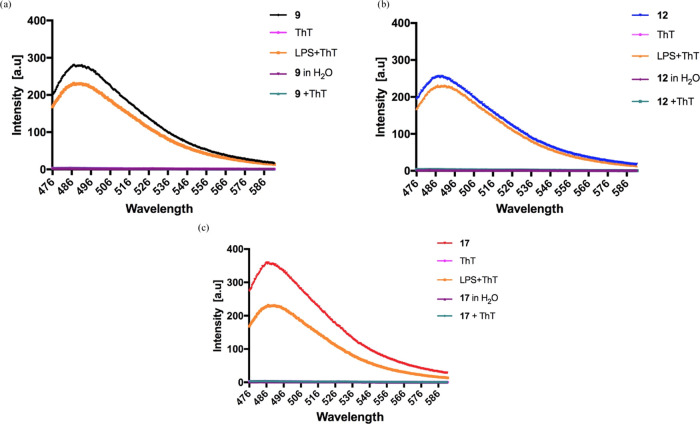
Aggregate formation in the interaction between peptides **9** (a), **12** (b), and **17** (c) (20 μM) and LPS (1 mg/mL) monitored by ThT fluorescence (25 μM). Peptide alone and ThT alone were used as controls.

Generally, the reduction of the efficacy of AMPs might be correlated to a phenomenon of aggregation induced by LPS of the outer membrane of Gram-negative so that they are inactive because they are trapped and unable to cross the cell wall.^65^ The aggregation of peptides **9**, **12**, and **17** in the presence of LPS was studied to elucidate the different antimicrobial activity of cyclic peptides against Gram-negative strains. The peptides were incubated with LPS (1 mg/mL) at a concentration of 20 μM, and ThT (25 μM) was used as a fluorophore to measure aggregation. Interestingly, after the incubation with LPS, triazole-bridged peptide **17** showed a higher ability to aggregate revealed by a large enhancement of ThT fluorescence emission (Figure 5). Conversely, lactam-bridged peptide **12**, keeping a fair activity against Gram-negative bacteria, exhibited the lowest aggregation. Instead, the linear peptide **9** displayed an intermediate self-assembling ability in LPS at 20 μM, suggesting that the antimicrobial activity is correlated to its ability to disaggregate in LPS as already reported for the parent TL.^40^

### Membrane Fluidity

The lactam-bridged peptide **12**, endowed with an overall higher antibacterial activity and lower cytotoxicity compared to **9**, was further investigated to unravel its mechanism of action. A membrane fluidity study was performed using Laurdan, a probe with the capacity to insert into membranes and to distribute between liquid phases,^66^ changing its emission spectrum.^67^ In particular, the maximum emission wavelength of Laurdan is 490 nm when phospholipids are in a disordered phase and it shifts to 440 nm when lipids are in a more packed phase. The generalized polarization (GP) parameter was used to measure quantitatively the emission shift of the Laurdan probe.^68^ The variation of fluidity membrane in the presence of peptide **12** was evaluated at 5 and 30 μM using LUVs mimicking Gram-positive and Gram-negative membranes. LUVs fluidity was evaluated before and after the addition of **12** and **9** at 25 °C. Before the addition of both peptides, emission of LUVs, mimicking both Gram-positive and Gram-negative membranes, indicated the presence of a disordered phase with a maximum at 490 nm (Table 3). The membrane fluidity did not significantly change in the presence of cyclic peptide **12** and its precursor **9** at a concentration of 5 μM. In fact, GP parameter indicated the presence of a disordered phase for both Gram-positive and Gram-negative liposomes (Table 3). Interestingly, a significant shift of the GP value toward more ordered membranes was observed at a concentration of 30 μM for both peptides.

**Table 3:** Membrane Fluidity Evaluation Using the GP Value

	GP_laurdan_		
cmpd	DOPG/CL	DOPG/CL + cmpd^a^	DOPG/CL + cmpd^b^
**9**	–0.32	–0.19	0.03
**12**	–0.32	–0.23	0.04

	GP_laurdan_		
cmpd	DOPG/DOPE/CL	DOPG/DOPE/CL + cmpd^a^	DOPG/DOPE/CL + cmpd^b^
**9**	–0.14	–0.1	0.05
**12**	–0.14	–0.07	0.1

a Concentration is 5 μM.

b Concentration is 30 μM.

### Ability of Peptide **12** to Induce Membrane Leakage

**Figure 6:**
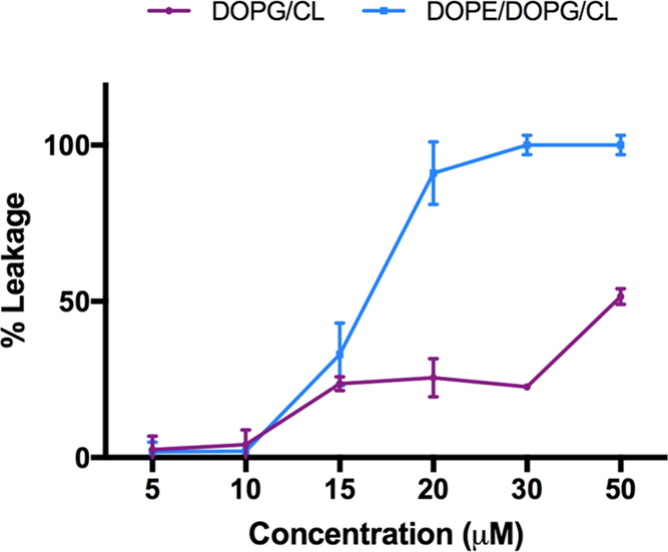
Ability of lactam-bridged peptide **12** to induce leakage of both LUVs mimicking Gram-positive (violet) and Gram-negative (blue) membranes.

The destabilization of membrane vesicles by **12** was explored measuring its effect on the release of encapsulated fluorophores in both LUVs mimicking Gram-positive and Gram-negative membranes. ANTS and DPX fluorophores were encapsulated in LUVs, and ANTS release was measured to evaluate the transient pore formation. Compound **12** induced leakage of LUVs mimicking Gram-positive and Gram-negative membranes, similarly to parent peptide **9** as elsewhere reported.^45^ Specifically, **12** showed a low leakage effect on LUVs mimicking Gram-positive membranes, while, interestingly, we observed a significant leakage ability of LUVs mimicking Gram-negative membranes, measured by a large increase of ANTs fluorescence depending on peptide concentration (Figure 6).

### Activity against MRSA ATCC and Time Kill Assay

**Figure 7:**
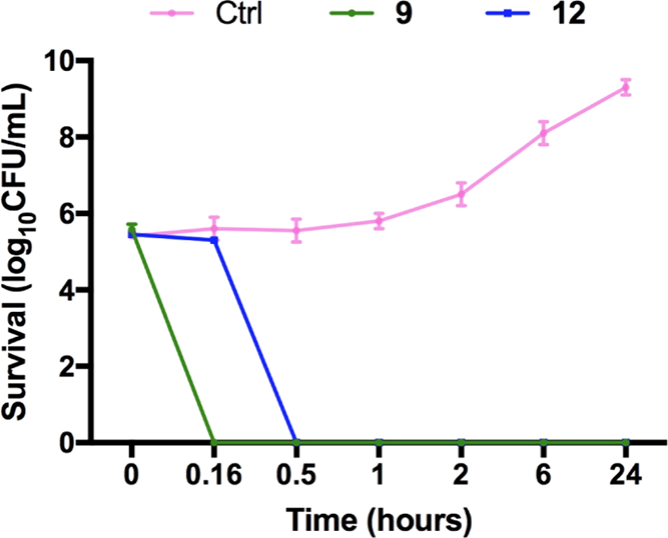
*In vitro* time kill assay. *S. aureus* ATCC 43300 was exposed to 12.5 μM peptide **12** and 25 μM peptide **9**, for 10 min, 30 min, 1 h, 2 h, 6 h, and 24 h. The inhibitory effect on bacterial growth was assessed by measuring the number of CFU obtained after the treatment.

From the results discussed until now, peptide **12** displayed the best antimicrobial activity against *S. aureus* ATCC 25923. Thus, **12** was further tested against *S. aureus* ATCC 43300, a methicillin-resistant strain (MRSA), and compared with the linear precursor **9**. The MIC for peptide **12** was 12.5 μM, while for peptide **9**, it was 25 μM. The time kill assay results, presented in terms of the changes in the log_10_ CFU/mL of viable colonies, demonstrated that the MRSA strain was killed within 30 min after the addition of 12.5 μM peptide **12** (Figure 7). The rapid reduction in the number of bacterial cells indicated that the killing of this strain by peptide **12** occurred immediately. No growth was observed after 24 h. Peptide **9** was bactericidal as well, by killing MRSA within 10 min, but at a higher concentration (25 μM).

### Antibiofilm Activity

**Figure 8:**
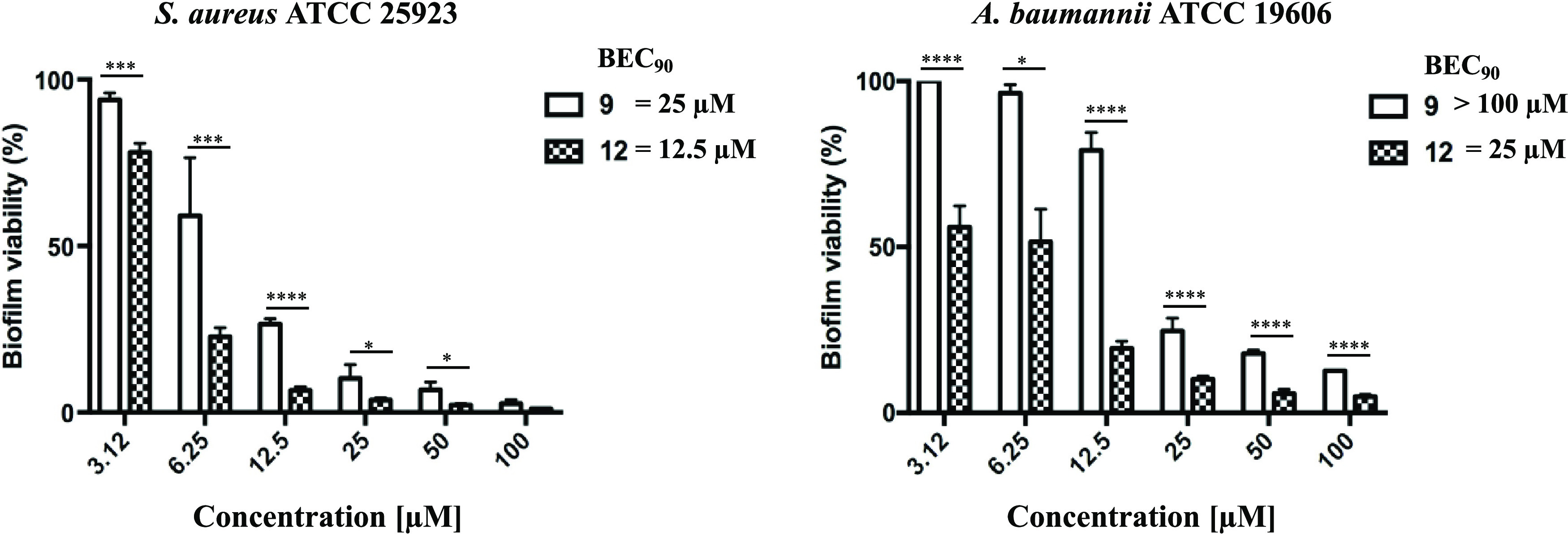
Antibiofilm activity of **9** and **12** against preformed biofilm of *S. aureus* ATCC 25923 and *A. baumannii* ATCC 19606 after 2 h of peptide treatment. Biofilm viability was determined as indicated in the Materials and General Procedures section and expressed as a percentage compared to that of untreated samples (100%). Values are the mean of at least three replicates ± SEM. Data are considered to be statistically significant as follows: *p < 0.05; ***p < 0.001; ****p < 0.0001. Biofilm eradication concentration 90, BEC_90_, was defined as the concentration able to reduce at least 90% of biofilm cells.

Considering the innate tendency of *S. aureus* and *A. baumannii* to form sessile communities (biofilms) that are much more difficult to eradicate, peptide **12** was further characterized for its ability to kill biofilm cells in comparison with peptide **9**. As shown in Figure 8, already after 2 h of treatment, peptide **12** was able to reduce more than 90% of viable *S. aureus* biofilm cells (with respect to untreated samples) from 100 to 12.5 μM. The same potent activity was shown by peptide **12** against preformed *A. baumannii* biofilm from 100 to 25 μM. In contrast, a significant weaker antibiofilm activity was shown by the parent peptide **9**, with the only exception of 100 μM against *S. aureus* biofilm. Note that for both peptides **9** and **12**, the concentration able to reduce at least 90% of biofilm cells (Biofilm Eradication Concentration 90, BEC_90_) against *S. aureus* resulted 4-fold higher than MIC (**9**: BEC_90_, 25 μM; **12**: BEC_90_, 12.5 μM). In comparison, against *A. baumannii*, peptide **9** had BEC_90_ > 100 μM, while for peptide **12**, BEC_90_ was 25 μM (8-fold higher than MIC).

### Human Serum Stability

**Figure 9:**
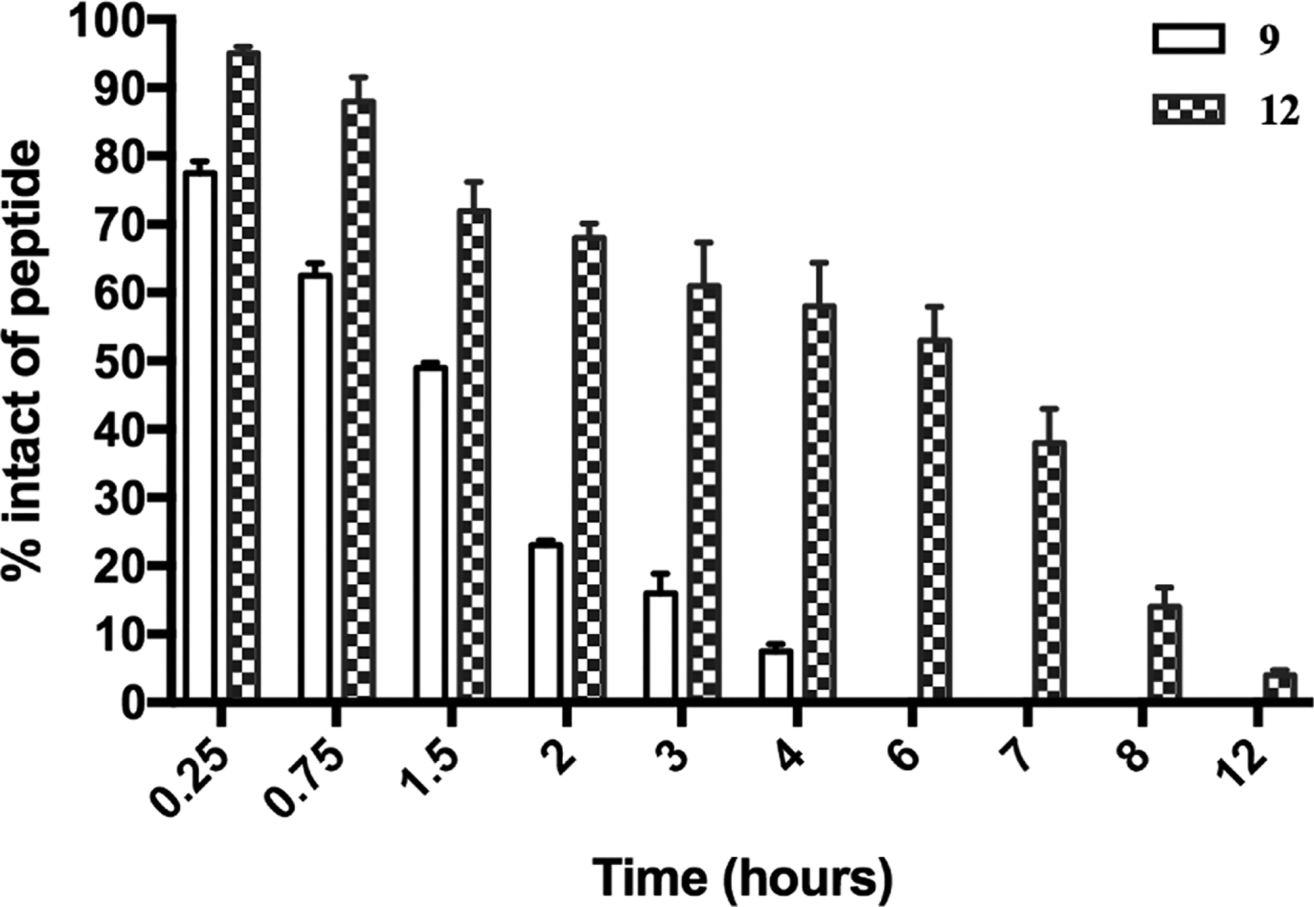
Percentage of intact lactam-stapled peptide (**12**) and its linear precursor (**9**) by an incubation with 90% human serum at 37 ± 1 °C at different time intervals (0.25, 0.75, 1.5, 2, 3, 4, 6, 7, 8, and 12 h).

The effects of macrocyclization on peptide stability were established by subjecting both the linear (**9**) and stapled peptide (**12**) to proteolytic degradation. Peptides were incubated with 90% fresh human serum at 37 ± 1 °C within 12 h.^69^ The percentage of intact peptide was monitored by calculating the peak area of the chromatogram from RP-HPLC analysis. As reported in Figure 9, the lactam-stapled peptide (**12**) showed a higher proteolytic stability than linear precursor. While **9** was completely degraded within 6 h, at the same time interval, **12** was still 50% intact at the same time interval and appears to be fully degraded only after 12 h (*t*_1/2_ ∼ 6 h).

### Structure Determination of Lactam-Bridged Peptide **12** by NMR Spectroscopy

The most active peptide **12** was analyzed by solution NMR spectroscopy. Since peptide **12** showed broad proton signals in SDS solution, a complete series of 1D and 2D spectra were acquired in SDS/DPC 9:1, where **12** shows relatively sharp and not overlapping signals. Complete ^1^H NMR chemical shift assignments were effectively achieved according to the Wüthrich procedure (Table S2).^70^ Peptide **12** exhibited NMR spectral features pointing to helical propensity in the micellar solution. Upfield shift of the Hα NMR signals and temperature coefficients of many amide protons (Table S2) and diagnostic NOEs (Table S3) indicated that many residues in the central and C-terminal region are in a helical conformation.

**Figure 10:**
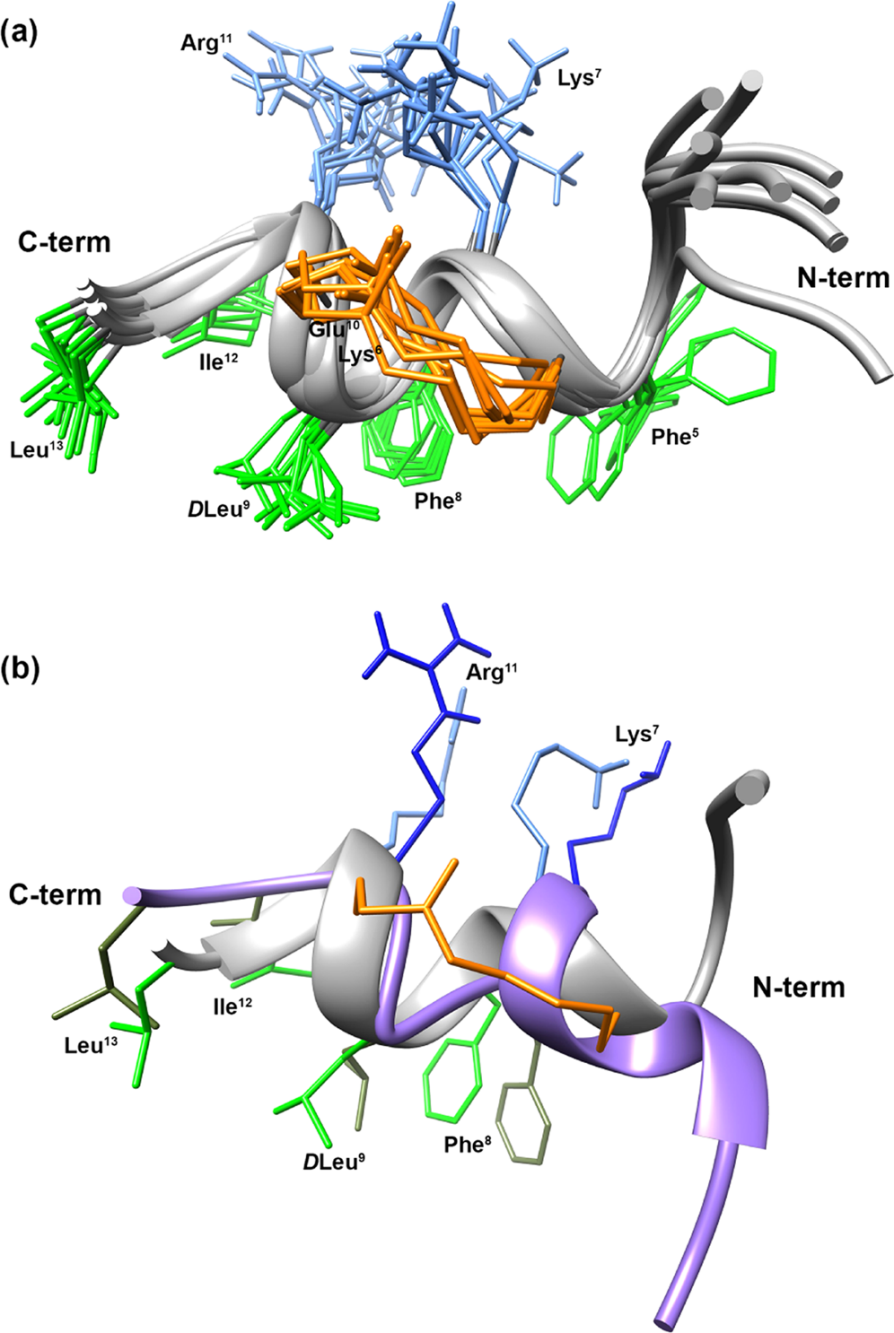
(a) NMR-derived 10 lowest-energy structures of peptide **12** (PDB ID 7OSD). (b) Superimposition of the lowest-energy conformers of peptide **12** (gray cartoon) and **9** (purple cartoon). Helical regions are depicted as ribbon. Side chains of hydrophobic and hydrophilic residues in the C-terminal regions are reported in light (**12**) or dark (**9**) green and cyan (**12**) or blue (**9**), respectively. Bridge atoms are reported in orange.

Structure calculation of peptide **12** based on NMR constraints gave an ensemble of 20 structures (Figure 10a) satisfying the NMR-derived constraints (violations smaller than 0.10 Å). An inverse γ-turn centered on the Pro^3^ can be observed at the N-terminus followed by an α-helix from Phe^5^ to Leu^13^. Notably, d-Leu^9^ shows a negative value for the φ angle (−55 ± 4), perfectly fitting an α-helix, despite its d-configuration. Backbone is well defined within the helical region with RMSD = 0.29 Å (residues 5–13). Also, the side chains are well defined apart from those of Phe^1^, which is very flexible, and Phe^5^, which shows both *trans* and *gauche* orientations. The peptide assumes an amphipathic structure with a positive side (Figure 10a, top) and a hydrophobic side (Figure 10a, bottom). Interestingly, the lactam bridge lies approximately in the middle of the two opposite featured sides.

## Discussion

The development of novel classes of antibiotics is crucial to tackle the forthcoming global issues of infections caused by critical pathogens and related antibiotic resistance.^28^ To cope with this challenge, AMPs may serve as a class of valuable antimicrobial agents due to their alternative mechanisms of action, including membranolytic activity, against several Gram-positive and Gram-negative bacteria.^71^ To fine-tune the therapeutic index of temporins, known to be related to the peptide secondary structure,^72,73^ an intramolecular macrocyclization peptide strategy has been applied. Specifically, novel cyclic peptidomimetic derivatives were designed and synthesized by means of different staple architectures in the C-terminus region of [Pro^3^,dLeu^9^]TL (**9**).^45^ Side-chain-to-side-chain lactam and 1,4-triazolic stapling strategies were employed to build up the cyclic analogues **10**–**23**. The cyclizing amino acids were introduced at various i,i+4 and i,i+7 positions, to primarily detect the suitable stapling positions that would result in a proper conformational stabilization and an optimal antimicrobial profile. During the incorporation of Lys^i^-Glu^i+4/7^ and Pra^i^-Lys(N_3_)^i+4/7^ pairs, positively charged residues, i.e., Lys^7^ and Arg^11^, were considered indispensable for the antimicrobial activity due to their role of driving force in the negatively charged membrane phospholipids interactions. Also, d-configuration in position 9 was still kept unaltered for cyclizing amino acids replacing the original d-Leu (peptides **10**, **13**, **15**, and **18**).

By screening peptides **10**–**23** for antimicrobial activity against selected Gram-positive (*S. aureus* ATCC 25923, *S. epidermidis* ATCC 12228, *B. megaterium* Bm11) and Gram-negative (*E. coli* ATCC 25922, *P. aeruginosa* ATCC 27853, *A. baumannii* ATCC 19606) strains (Table 1), it was clear that the cyclization position was the most crucial element, regardless of the side-chain tethering strategy used. In particular, when the aromatic amino acids such as Trp^4^, Phe^5^, and Phe^8^ were replaced with cyclizing amino acids, the resulting cyclic analogues were characterized by a dramatic loss of activity. In fact, lactam-bridged peptides **11**, **13**, and **14** resulted to be completely inactive against both Gram-positive (MIC, 50 or MIC > 100 μM) and Gram-negative (MIC > 100 μM). The 1,4-triazolic bridged peptides **16**, **18**, and **19** were similarly inactive, with the sole exception of antimicrobial activity against the Gram-positive *B. megaterium* Bm11 strain, that is within the range 6.25–25 μM. These outcomes were in line with previous observations,^74^ since a subtraction of phenylalanine zipper (Phe^5^ and Phe^8^) is confirmed to be critical in determining the first-stage interactions with the bacterial membrane and mediating peptide self-assembling across the membrane.^72^ However, the presence of the triazole in the bridge of cyclic peptides **16**, **18**, and **19** was partially tolerated in the antimicrobial performance against *B. megaterium* Bm11 (Table 1). Probably, the triazolyl ring partially mimics the missing aromatic side chains enabling peptides to recover some activity. Peptides **10** and **15**, carrying both the cyclization between side chains in positions originally occupied by d-Leu^9^ and Leu^13^, were also inactive peptides, confirming that these are key residues in determining the hydrophobic character of the C-terminal region,^42,75^ and their substitution is detrimental. Conversely, the replacement of Ser^6^ and Gly^10^ amino acids with Lys^i^-Glu^i+4^ and Pra^i^-Lys(N_3_)^i+4^ pairs, to achieve lactam and triazole staples, respectively, produced two peptides, **12** and **17**, which perform as active antimicrobial agents, especially against Gram-positive bacteria. Particularly, both peptides exhibited significant activity against *S. aureus* ATCC 25923 (MIC, 3.12 μM), *S. epidermidis* ATCC 12228 (**12**: MIC,3.12 μM; **17**: MIC, 1.56 μM) and *B. megaterium* Bm11 (MIC, 1.56 μM), showing equal or lower MIC compared to the linear counterpart **9** (Table 1). Moreover, peptide **12** proved to be more active vs the Gram-negative strains compared to **17**, suggesting that lactam and triazole groups, albeit bioisosters, were specifically involved in the different antimicrobial spectrum of these cyclic temporin derivatives (**12** vs **17**). Interestingly, against human keratinocytes, peptide **12** also is less cytotoxic than **17** and **9** (Figure 2), even at a high concentration (50 μM). Conversely, a poor antimicrobial performance was observed for derivatives cyclized in i,i+7 positions (peptides **20**–**23**). In fact, among these, only weak activity against *B. megaterium* is retained by peptides **20**, **22**, and **23** (MIC, 12.5 μM), confirming the importance of residues Phe^5^, Ile^12^, and Leu^13^.

Upon the detection of Ser^6^ and Gly^10^ in the C-terminus of **9** as the optimal stapling positions, we carried out further stapling modifications to evaluate their impact on both the α-helical stabilization and biological activity, since it was above demonstrated that the type of side-chain-to-side-chain tethering may play a pivotal role in the antimicrobial spectrum of **9**-derived cyclic analogues. First, the switch of Lys and Glu amino acids in the sequence of **12** yielded peptide **24**, then two additional macrocyclizations, hydrocarbon (peptide **25**) and disulfide (peptide **26**) linkers, were employed. Of note, peptide **24** showed similar antimicrobial activity, compared to its parent peptide (**12**), with even enhanced efficacy against the Gram-positive *S. epidermidis* ATCC 12228 (MIC, 1.56 μM vs MIC, 3.12 μM). Interestingly, despite their similar antimicrobial profiles, **24** showed significantly stronger cytotoxicity compared to **12**. Peptide **25**, retaining the 21-membered cycle as lactam- and triazole-bridged peptide series (**10**–**19**), was completely inactive, while the disulfide linkage in peptide **26**, bearing a constrained 17-membered cycle, displayed reduced activity against several strains, including both Gram-positive and Gram-negative bacteria, compared to peptides **12** and **24**. Thus, among the newly generated compounds, **12** turned out to be the peptide endowed with an overall highest antibacterial activity and lowest cytotoxicity, up to a concentration of 50 μM, with respect to the reference peptide **9**.

Although we observed that the employment of additional linkers, unlike lactam, in the optimal positions of Ser^6^ and Gly^10^ led to less active compounds, we further investigated the correlation between the α-helical content and the biological behavior. Indeed, cyclic temporin analogues **12**, **17**, and **24**–**26** were evaluated for their conformational propensities in various environments (water, SDS, and DPC). In particular, CD measurements for these stapled peptides and linear reference compound **9** were performed (Figure 3 and Table 2). In water, all peptides showed an unordered conformation, except peptide **25**, bearing olefinic bridge, which displayed a tendency to form α-helix aggregates, probably determining the loss of activity. When structural analyses were carried out in SDS, all peptides showed a higher helical propensity, except for the disulfide-bridged peptide **26**. The latter rather showed a helical content in the same order of linear precursor **9**, confirming that the disulfide bridge does not induce a significant helicity in i,i+4 positions.^5,6^ Peptides **12** and **24**, instead, were the most structured in SDS solution with similar CD spectra and helix percentage (about 80%), followed by peptides **25** and **17** (∼60%). It is noteworthy that despite its high helical content in SDS, the stapled peptide **25** resulted completely inactive, probably due to its excessive self-aggregation in solution. Overall, comparing biological behaviors of peptides **12** and **17** to parent peptide **9** on Gram-positive strains, results corroborated a correlation between α-helical content and antimicrobial activity. However, the activity of peptide **26**, which shows a low helical content, indicates that other factors, such as amphipathicity, may also play an important role (see below). However, in line with previous SAR studies upon TL analogues, where a clear correlation of the cytotoxic effect with the helical propensity in zwitterionic DPC micelles is described,^43^ in our study, peptide **24**, displaying the highest helical percentage in this environment, showed the highest cytotoxic effect.

At this stage, our results did not completely elucidate the diverse antimicrobial performance of peptides **12** and **17** with respect to reference compound **9**, and therefore, we further investigated their interaction modality with cell membranes. We thus probed the ability of peptides to oligomerize in the presence of bacterial membrane using the ThT assay, as well as the conformation change induced by oligomerization was detected by CD analyses. In LUVs mimicking Gram-positive membranes (Figure 4a–c), while the linear peptide **9** oligomerized at a low concentration (10 μM), peptides **12** and **17** oligomerized at higher concentrations. All peptides showed the tendency to give helical aggregates as shown by CD spectra (Figure 4b). Interestingly, as depicted in Figure 4d–f, peptides **12** and **17** also showed the same behavior in the presence of LUVs mimicking Gram-negative membrane, oligomerizing in helical aggregates at a low concentration of 10 μM, like parent peptide **9**. Hence, we hypothesized that the reduced activity of peptide **17** compared to peptide **12** on Gram-negative strains might be caused by a peptide self-association in the presence of LPS. Indeed, after 1 h of incubation with LPS and ThT, the aggregation of triazole-bridged peptide **17** was more significant than lactam-bridged peptide **12** and linear precursor **9** disclosing a strong effect of aggregation on bactericidal activity of peptide **17** (Figure 5). Instead, we hypothesized that the linear peptide **9** is able to both aggregate and disaggregate in LPS as already reported for the native TL sequence; thus, its ability to self-assemble in LPS is not abolishing activity.^40^

We finally focused on peptide **12** with the aim to examine its mechanism of action by Laurdan and leakage assays in the presence of LUVs, mimicking both Gram-positive and Gram-negative membranes. By evaluating the fluidity of bilayer at 25 °C using Laurdan as fluorescent probe, lactam-bridged peptide **12** and its linear counterpart **9** showed the same trend, strongly influencing the membrane stability at a high concentration in the presence of Gram-positive and Gram-negative LUVs (Table 3). In addition, the ability of lactam-bridged **12** to induce the pore formation was investigated using ANTS and DPX as fluorescent probes. Peptide **12** showed the same mechanism of action of linear peptide **9**, as elsewhere reported.^76^ Peptide **12** caused a low leakage on LUVs mimicking the membrane of Gram-positive bacteria, whereas a strong leakage effect was observed on LUVs mimicking Gram-negative bacterial membranes. This finding is in line with the capability of the peptide to kill MRSA bacterial cells, as demonstrated by time kill assay (Figure 7), and to reduce the viability of *S. aureus* and *A. baumannii* biofilms. Indeed, after the addition of peptide **12** at 12.5 μM, MRSA cells were completely killed within 30 min. The same bactericidal effect was observed for peptide **9** within 10 min albeit at the higher concentration of 25 μM. Moreover, peptide **12** showed significantly higher antibiofilm activity than the reference peptide **9** against *S. aureus* with a BEC_90_ corresponding to 12.5 μM vs 25 μM, respectively. The same potent antibiofilm activity was also detected against *A. baumannii* biofilm, with BEC_90_ corresponding to 25 μM for peptide **12** vs >100 μM for reference peptide **9** (Figure 8).

Active and safe peptide **12** was further investigated by solution NMR. NMR analysis followed by structure calculation gave the conformer ensemble shown in Figure 10a. The obtained 3D structure can give a tentative explanation of the antimicrobial activity observed for **12** and, considering that bridge is positioned at the same position, also for the other derivatives stapled in Ser^6^–Gly^10^. Peptide **12** displayed an amphipathic α-helix along residues 5–13. The bridge is located just in between the two opposite faces of the amphipathic structure. Hence, it is conceivable that a partially polar structure, as an amide (**12**, **24**), triazole (**17**), or disulfide (**26**), should not perturb the amphipathicity while a nonpolar hydrocarbon staple (**25**) will destroy it. Hence, inactivity of **25** can be ascribed to its loss of amphipathicity, in addition to its aggregation tendency. These findings confirm that an amphipathic structure more than the α-helix itself is mandatory for the antimicrobial activity of temporin-derived peptides. Comparing the NMR structure of peptides **9** and **12** (Figure 10b), the helical region of peptide **12** involves more residues (∼9 in **12** vs ∼5 in **9**), as expected from the design strategy. However, the corresponding side chains within the region 7–13 show almost the same orientation preserving an amphipathic structure. In contrast, the N-terminal regions point to different directions, suggesting a putative correlation with the reduced cytotoxic effect observed for **12** compared to **9**, as the orientation of this region proved to be crucial in determining the cytotoxic effect of TL and its analogues.^43,44^

Despite several advantages in the use of peptides as drugs, their low proteolytic stability still remains a hard challenge to face. In general, a macrocyclization established by an α-helix inducer, such as lactam linker, might provide to peptide a high proteolytic resistance in comparison to linear precursor. Based on this consideration, we explored the effects of macrocyclization on peptide serum stability revealing that the stapled peptide **12** had higher proteolytic stability than its linear precursor (Figure 9). In fact, ∼40% of linear peptide was degraded within 45 min, whereas nearly ∼70% of lactam-stapled peptide (**12**) was still intact after 90 min of incubation. Interestingly, a percentage of ∼50% of peptide **12** was detected within 6 h of treatment with human serum, while at the same time, interval peptide **9** was completely degraded. These results showed that macrocyclization can be an efficacious strategy to reduce protease susceptibility typical in AMPs.

## Conclusions

We applied stapling techniques by means of lactam, 1,4-substituted [1,2,3]-triazole, hydrocarbon and disulfide linkers, to synthesize the first library of cyclic temporin isoforms. Our strategy resulted in the identification of specific positions within **9** (Ser^6^ and Gly^10^), which allowed the introduction of cyclizing amino acids without affecting the antimicrobial activity. In particular, the lactam bridge in **12** turned out to be effective for the generation of a new therapeutic agent with both antibacterial and antibiofilm activities. Screening diverse cyclization linkers shed light on the impact of the α-helical content stabilization on the biological behavior. Notably, we found that the olefinic bridge (e.g., **25**) resulted in the formation of α-helix aggregates, while the disulfide bridge (e.g., **26**) was not able to induce strong helicity so that their antimicrobial activities were impaired. Peptide **24**, albeit possessing antimicrobial activity similar to that of **12**, showed higher cytotoxicity, confirming the high degree of helicity in mammalian membranes environment as detrimental for human cell viability. Also, the aggregation phenomenon occurring for **17** in the presence of LPS was crucial for its reduced activity against Gram-negative strains compared to **12**. Therefore, the novel cyclic analogues described in this study provided the means to expand our knowledge on the use of intramolecular cyclization chemistry for antimicrobial peptides and offered more insights into the mechanism of action of these peculiar AMPs derivatives within the family of temporins. Finally, our ongoing efforts to improve drug-like features of these agents led to the discovery of a new cyclic antimicrobial and antibiofilm peptide compound, **12**, suitable for the development of next cyclic analogues of this class of AMPs.

## Experimental Section

### Materials and General Procedures

The bacterial strains used in the antimicrobial assays were the Gram-negative *E. coli* ATCC 25922, *P. aeruginosa* ATCC 27853, *A. baumannii* ATCC 19606, the Gram-positive *S. aureus* ATCC 25923, *S. epidermidis* ATCC 12228, and *B. megaterium* Bm11. Methicillin-resistant *S. aureus* (MRSA) ATCC 43300 was obtained from Culture Collection Center of School of Basic Medical Sciences of Lanzhou University (China).

The *N*^α^*-*Fmoc-protected conventional amino acids, Fmoc-Phe, Fmoc-Val, Fmoc-Pro, Fmoc-Trp(Boc), Fmoc-Ser(*t*Bu), Fmoc-Lys(Boc), Fmoc-Gly, Fmoc-Leu and Fmoc-d-Leu, Fmoc-Ile, and Fmoc-Arg(Pbf) were acquired from GL Biochem Ltd. (Shanghai, China). Fmoc-Lys(Alloc), Fmoc-d-Lys(Alloc), Fmoc-Glu(OAll), Fmoc-d-Glu(OAll), Fmoc-Pra, Fmoc-d-Pra, Fmoc-Lys(N_3_), Fmoc-d-Lys(N_3_), *N,N*-diisopropylethylamine (DIEA), piperidine, and trifluoroacetic acid (TFA) were purchased from Iris-Biotech GMBH. Coupling reagents such as *N,N,N*′*,N*′-tetramethyl-O-(1H-benzotriazol-1-yl) uranium hexafluorophosphate (HBTU) and 1-hydroxybenzotriazole (HOBt) were commercially obtained by GL Biochem Ltd (Shanghai, China). The rink amide resin with a loading substitution of 0.72 mmol/g was purchased by Iris-Biotech GMBH. Anhydrous solvents [*N,N*-dimethylformamide (DMF), dichloromethane (DCM), 1,2-dichloroethane (DCE)], morpholino-carbenium hexafluorophosphate (COMU), and ethyl cyano(hydroxyimino)acetate (Oxyma) were obtained from Sigma-Aldrich/Merck. All useful reagents for the synthesis of cyclic peptides, such as 1,3-dimethylbarbituric acid (NDMBA), tetrakis(triphenylphosphine)palladium(0) [Pd(PPh_3_)_4_], ascorbic acid, copper (I) iodide, 2,4,6-collidine, 2,2,2-trifluoroethanol, were purchased from Sigma-Aldrich/Merck. (*S*)-Fmoc-2-(4′-pentenyl)alanine-OH was obtained by Okeanos Tech. Co. Moreover, peptide synthesis solvents, *N,N*-dimethylformamide (DMF), dichloromethane (DCM), diethyl ether (Et_2_O), water, and acetonitrile (MeCN) for HPLC, were of reagent grade acquired from commercial sources (Sigma-Aldrich and VWR) and used without further purification.

Phospholipids: 1,2-dioleoyl-sn-glycero-3-phosphoethanolamine (DOPE), 1,2-dioleoyl-sn-glycero-3-phospho-(1′-rac-glycerol) sodium salt (DOPG), and cardiolipin (CL) sodium salt (Heart, Bovine) were purchased from Avanti Polar Lipids (Birmingham, AL), Phosphate-buffered saline (PBS) tablets were bought by Life Technologies Corporation. 8-Aminonaphtalene-1,3,6-trisulfonic acid, disodium salt (ANTS), and *p*-xylene-bis-pyridinium bromide (DPX) were purchased from Molecular Probes. MTT [3-(4,5-dimethylthiazol-2-yl)-2,5-diphenyltetrazolium bromide], Triton X-100, Thioflavin T, 6-dodecanoyl-*N,N*-dimethyl-2-naphthylamine (Laurdan), sodium dodecyl sulfate (SDS), and dodecylphosphocholine (DPC) were purchased from Sigma-Aldrich/Merck. Human serum from human male AB plasma, USA origin, sterile-filtered, was obtained from Sigma-Aldrich/Merck.

Analytical UHPLC (Shimadzu Nexera Liquid Chromatograph LC-30AD) analyses to assess critical synthetic steps as well as the purity of final compounds **10**–**26** were performed on a Phenomenex Kinetex reversed-phase column (C18, 5 μm, 100 Å, 150 × 4.6 mm) with a flow rate of 1 mL/min using a gradient of MeCN (0.1% TFA) in water (0.1% TFA), from 10 to 90% over 20 min, and UV detection at 220 and 254 nm. Purification of peptides **10**–**26** was performed by RP-HPLC (Shimadzu Preparative Liquid Chromatograph LC-8A) equipped with a preparative column (Phenomenex Kinetex C18 column, 5 μm, 100 Å, 150 × 21.2 mm^2^) using linear gradients of MeCN (0.1% TFA) in water (0.1% TFA), from 10 to 90% over 30 min, with a flow rate of 10 mL/min and UV detection at 220 nm. Final products were obtained by lyophilization of the appropriate fractions after removal of the MeCN by rotary evaporation. All compounds examined for biological activity were purified to >97% (Figures S1–S17), and the correct molecular ions were confirmed by HRMS spectrometer (LTQ Orbitrap) (see Table S1).

### Peptide Synthesis

#### Synthesis of Resin-Bound Linear Peptide Sequences

The peptide sequences were assembled stepwise by embracing the Fmoc-based US–SPPS method.^53^ In particular, the Fmoc Rink amide resin (0.1 mmol; 0.72 mmol/g as loading, 100–200 mesh as particle size) was placed into a 10 mL polypropylene tube (ISOLUTE SPE filtration column by Biotage, Uppsala, Sweden) equipped with a filter (ISOLUTE frits, 20 μm porosity polyethylene frits by Biotage, Uppsala, Sweden), stopper, and top cap, and swollen in DMF for 20 min. The Fmoc group from the rink amide linker was first removed by adding a 20% piperidine in DMF solution and by placing the tube reactor in an ultrasonic bath with the reaction mixture not exceeding the water level (0.5 + 1 min). After each step, filtering and washings of the resin were executed (3 × 2 mL of DMF; 3 × 2 mL of DCM). Then, the coupling reactions were carried out by treatment with a solution of the Fmoc-amino acid (2 equiv), COMU (2 equiv), Oxyma (2 equiv), and DIEA (4 equiv) in DMF, and exposing the resin to the ultrasonic irradiation for 5 min. To monitor both Fmoc deprotection and coupling reactions, the colorimetric Kaiser or chloranil tests were used for the detection of solid-phase bound primary and secondary amines, respectively.

#### On-Resin Lactamization

The synthesis of side-chain-to-side-chain lactam-bridged peptides **10**–**14** continued on solid phase. The peptide sequences were treated for the selective removal of allyl-based protecting groups of lysine and glutamic residues, according to procedures elsewhere described.^54^ In particular, the resins were washed with DCM (3 × 2 mL), suspended in a solution of Pd(PPh_3_)_4_ (0.15 equiv) and NDMBA (3 equiv) in dry DCM/DMF (3:2 v/v), and gently shaken for 1 h under Ar. The resin was filtered, washed with DMF (3 × 2 mL) and DCM (3 × 2 mL), and dried. Then, such allyl deprotection procedure was repeated for a second time. Finally, an additional washing with 0.5% sodium *N,N*-diethyldithiocarbamate solution in DMF (30 min × 2) was made, and the complete removal of the allyl groups was ascertained by LC–MS analysis of the residue from the cleavage of an aliquot of resin [5 mg treated with 1 mL of TFA/TIS/H_2_O (95:2.5:2.5, v/v/v)]. The released amine and carboxylic acid were thus coupled using PyAOP (2 equiv), HOAt (2 equiv), and DIEA (4 equiv), dissolved in DMF/DCM (1:1, v/v). The reaction mixture was placed in an automated shaker for 12 h at rt, and the conversion to the cyclic product was monitored by LC–MS analysis of the residue from the cleavage of an aliquot of resin (5 mg). Finally, the N-terminal Fmoc group was removed and the peptidyl resin was washed three times with DCM and dried in vacuo.

#### On-Resin “Click” Reaction

Copper (I)-catalyzed azide–alkyne cycloaddition on solid phase (CuAAC-SP) was performed to obtain the selective formation of 1,4-disubstituted [1,2,3]-triazole regioisomers of peptides **15–19**. The side-chain-to-side-chain CuAAC-SP cyclization was thus performed between the alkyne and azido groups inside the chain of Pra and Lys(N_3_) residues, respectively. Prior elongation, after the coupling of Fmoc-Pra, the CuAAC-SP was carried out.^49,56^ Copper iodide (1 equiv) and l-ascorbic acid (3 equiv) were dissolved in DMF (3 mL for 0.1 mmol of resin) and then 2,4,6-collidine (5 equiv) and DIEA (10 equiv) were added. The resulting solution was added to the resin, and the SPPS reactor was gently shaken for 1 h at rt. After this first cycle, the resin was washed with DCM (3 × 2 mL) and DMF (3 × 2 mL) and the CuAAC-SP procedure was repeated twice. The formation of 1,4-triazole bridge was monitored by retention time shifts observed by LC–MS analyses of a residue from the cleavage of an aliquot of resin [5 mg treated with 1 mL of TFA/TIS/H_2_O (95:2.5:2.5, *v/v/v*)]. Upon the quantitative conversion, the synthesis proceeded by US–SPPS protocols until the Fmoc removal from Phe^1^.

#### Ring-Closing Metathesis (RCM)

During the construction of the linear peptide sequence of **25**, the (*S*)-Fmoc-protected (*S*)-2-(4-pentenyl)alanine residues (Fmoc-S_5_-OH) were introduced for the formation of the olefin bridge. The RCM reaction was carried out on the resin-bound and fully protected peptide.^57^ In particular, the resin was washed with DCM (3 × 1 min) and DCE (3 × 1 min). Then, the resin was treated with a fresh 6 mM (3 mL) of the Grubbs’ first-generation catalyst in DCE (20 mol % relative to the resin substitution). The resulting suspension was gently agitated for 2 h with constant bubbling of nitrogen. Solvent evaporation was checked setting a low nitrogen pressure for bubbling. After the first round, the RCM reaction was repeated twice under the same condition. In the end, the resin was washed with DCE (3 × 1 min) and DCM (3 × 1 min) and then dried under a stream of nitrogen. The conversion in olefin bridge was monitored by LC–MS. Finally, the *N*-terminal Fmoc group was removed prior cleavage.

#### Off-Line Cysteine Oxidation and Disulfide Formation

The formation of disulfide bridge between two cysteine residues in **26** was achieved by *N*-chlorosuccinimide (NCS) oxidation in solution.^58^ Thus, upon the achievement of linear peptide sequence, this was prior cleaved by treatment with a cocktail of TFA/TIS/H_2_O (95:2.5:2.5 v/v/v) for 3 h, at rt. Then, the crude peptide (0.1 mmol) with purity >85% was dissolved in H_2_O (0.5 mM) and NCS (1 equiv) solution in H_2_O (5 mL) was added under stirring. The mixture was shaken for 15 min at rt and then lyophilized.

#### Peptide Cleavage

Peptides **10–26** were released from the resin and the protecting groups cleaved simultaneously by treatment with a cocktail of TFA/TIS/H_2_O (95:2.5:2.5 *v/v/v*) for 3 h at rt. Then, the resin was removed by filtration and crude peptides were precipitated and washed with chilled Et_2_O twice and were separated by centrifugation (2 × 15 min, 6000 rpm). The supernatants were removed, while the crudes were dried in vacuo and then dissolved in 10% MeCN in H_2_O to be purified by RP-HPLC.

### Biology

#### Antimicrobial Susceptibility Testing

The determination of MICs was conducted as previously reported.^61^ Aliquots of 50 μL of bacterial culture appropriately diluted were added to each well of a 96-well plate to reach a final cell density of 1 × 10^6^ CFU/mL and a final peptide concentration ranging from 100 to 0.78 μM (final volume 100 μL). The plates were incubated at 37 °C for 16–18 h, and the MIC was defined as the lowest concentration of drug that causes a total inhibition of microbial growth.

#### Mammalian Cells

The human immortalized keratinocytes HaCaT cells (AddexBio, San Diego, CA) were cultured in Dulbecco’s modified Eagle’s medium containing 4 mM glutamine (DMEMg), supplemented with 10% heat-inactivated fetal bovine serum (FBS) and 0.1 mg/mL of penicillin and streptomycin, at 37 °C and 5% CO_2_, in 25 or 75 cm^2^ flasks.

#### Cytotoxicity Assays

The quantification of cell viability was performed by the in vitro MTT [3-(4,5-dimethylthiazol-2-yl)-2,5-diphenyltetrazolium bromide] assay, as previously reported,^77^ measuring the conversion of the soluble yellow dye MTT to insoluble purple formazan by mitochondrial dehydrogenases. More precisely, 4 × 10^4^ HaCaT cells, suspended in DMEMg supplemented with 2% FBS without antibiotics, were plated in each well of the 96-well plate. After overnight incubation at 37 °C and 5% CO_2_, the medium of each well was replaced by DMEMg containing the peptide at different concentrations, as indicated. Controls were HaCaT cells not treated with the peptide. After 2 and 24 h of incubation at 37 °C and 5% CO_2_, the medium was discarded; 0.5 mg/mL of MTT in Hank’s buffer was added to each well, and the plate was incubated for further 4 h. Acidified isopropanol was then added to dissolve formazan crystals, and the absorbance of each well at 570 nm was measured using a microplate reader (Infinite M200; Tecan, Salzburg, Austria). The purple color intensity is directly proportional to the number of viable cells, which was expressed as a percentage compared to that of untreated control cells (100%).

#### Killing Rate

Time kill assay was carried out only for peptide **12** and peptide **9** on *S. aureus* MRSA ATCC 43300, as previously described,^78^ with minor modifications. Bacterial suspension (10^6^ CFU/mL) was added to microplates along with peptide **12** and peptide **9** at the MIC. The plates were incubated at 37 °C on an orbital shaker at 120 rpm. Viability assessments were performed at 10 min, 30 min, 1 h, 2 h, and 24 h by plating 0.01 mL of undiluted and 10-fold serially diluted samples onto Mueller–Hinton plates in triplicate. After overnight incubation at 37 °C, bacterial colonies were counted and compared with counts from control cultures.

#### Antibiofilm Activity

Preformed biofilms of *S. aureus* ATCC 25923 and *A. baumannii* ATCC 19606 were obtained as previously reported.^77^ Microbial cultures were grown in Luria-Bertani broth (LB) at 37 °C to an optical density (OD) of 0.8 (λ = 590 nm) and then diluted in LB to a cell density of 1 × 10^6^ colony-forming units (CFUs)/mL. Aliquots of 100 μL were dispensed into the wells of a 96-well plate, which was incubated for 20 h at 37 °C to allow biofilm formation. The medium containing planktonic cells was then aspirated from the wells and washed twice with 150 μL of phosphate-buffered saline (PBS) to remove any nonadherent cells. Each well was filled with PBS supplemented with twofold serial dilutions of peptides **9** and **12** (from 100 to 3.12 μM), and the plates were incubated for 2 h at 37 °C. After treatment, each well was rinsed twice with PBS, as previously described. Then, 150 μL of MTT solution (0.5 mg/mL) were added to each well to evaluate biofilm cell viability. The plates were incubated at 37 °C for 4 h in the dark, and the reaction was stopped by adding sodium dodecyl sulfate (SDS) (at a final concentration of 5% v/v). The absorbance of each well was recorded at 570 nm using a microplate reader (Infinite M200; Tecan, Salzburg, Austria), and the percentage of biofilm cells viability was calculated with respect to the untreated samples (100%).

### Antimicrobial Mechanism of Action Studies

#### Laurdan GP Value

Laurdan is extensively used as a fluorescent probe in membrane fluidity study.^79^ Laurdan emission spectra encapsulated in large unilamellar vesicles (LUVs) are centered at 490 nm when the lipids are in a disordered phase and the emission shifts to 440 nm when the lipids are in a more packed phase. The preparation of LUVs was performed using the extrusion method.^79^ To mimic the lipid composition of Gram-positive and Gram-negative membranes, we used LUVs made of DOPG/CL (58/42 ratio in moles) and DOPG/DOPE/CL (63/23/12 ratio in moles), respectively. First, stock solutions of lipids in chloroform were prepared and the assay was performed using a final lipid concentration of 100 μM. Laurdan (1 μM) was added to chloroform/lipids solution before the evaporation of organic solvent. The mixture of lipids and probe was dried under nitrogen gas stream and freeze-dried overnight. Then, lipid film with Laurdan was hydrated with PBS 1× buffer, pH = 7.4, vortexed for 1 h, and freeze-thawed six times and extruded 10 times through polycarbonate membranes with 0.1 μm diameter pores, obtaining LUVs with encapsulated Laurdan. Stock solutions of 2 mM of linear peptide **9** and lactam-bridged analogue **12** were prepared by dissolving peptides in water. The influence of peptides on membrane fluidity was evaluated at 5 and 30 μM. The peptide was added to LUVs + Laurdan probe, and after 10 min, the fluorescence spectra were recorded using a 1 cm path length quartz cell, thermostated at 25 °C. To measure the Laurdan emission shift and to quantify the variation of membrane fluidity, we calculated the generalized polarization (GP) as GP = (*I*_440_ – *I*_490_)/(*I*_440_ + *I*_490_).

#### Thioflavin T Fluorescence

The fluorescent dye Thioflavin T (ThT) is widely used to quantify peptide aggregation in the presence of bacterial membranes represented by LUVs (Gram-positive, DOPG/CL, 58/42 ratio in moles; Gram-negative DOPG/DOPE/CL, 63/23/12 ratio in moles). Once lipid films were prepared as described above, they were hydrated with 100 mM NaCl, 10 mM Tris-HCl, 25 mM ThT buffer, pH = 7.4, vortexed for 1 h, freeze-thawed six times, and extruded 10 times through polycarbonate membranes with 0.1 μm diameter pores.^80^ Peptides were dissolved in water to have a stock solution of 2 mM. Peptide aggregation was quantified by treating LUVs with peptide concentrations of 5, 10, 15, 20, 30, and 50 μM. Fluorescence was measured before and after the addition of peptide at 25 °C using a Varian Cary Eclipse fluorescence spectrometer. ThT fluorescence emission was measured at 482 nm (slit width, 5 nm) after excitation at 450 nm (slit width, 10 nm). In the presence of aggregated peptides, ThT combines with them giving an excitation maximum at 450 nm and an increased emission at 482 nm.^62^ Peptide aggregation was calculated as %A = (*F*_f_ – *F*_0_)/(*F*_max_ – *F*_0_) × 100, where *F*_f_ indicates the value of fluorescence after peptide addition, *F*_0_ is the initial fluorescence in the absence of peptide, and *F*_max_ is the fluorescence maximum obtained immediately after peptide addition.

#### Peptide Aggregation in LPS

Peptides **9**, **12**, and **17** at a concentration of 20 μM were incubated with LPS (1 mg/mL) and ThT (25 μM) for 1 h for a final volume of 300 μL. After 1 h, we evaluated the peptide aggregation measuring ThT fluorescence emission at 482 nm (slit width, 5 nm) after excitation at 450 nm (slit width, 10 nm). ThT spectra at 25 μM, and peptide alone at 20 μM were carried out as control.

#### ANTs/DPX Leakage Assay

Liposomes mimicking Gram-positive and Gram-negative membranes were loaded with ANTs and DPX fluorescent probes for monitoring their leakage induced by lactam-bridged peptide **12**. The dry lipid films (*C*_f_ = 100 μM) were dissolved with a solution of ANTs (12.5 mM) and DPX (45 mM) in water (2 mL) and then lyophilized overnight. The lipid films with encapsulated ANTS and DPX were hydrated with PBS 1× buffer and treated to obtain LUVs using the extrusion method.^81^ Then, nonencapsulated ANTs and DPX were separated from liposomes by gel filtration using a Sephadex G-50 column (1.5 cm × 10 cm) at room temperature. Initially, liposomes contain both ANTs and DPX, and DPX efficiently quenches ANT fluorescence by collisional transfer.^82^ When LUVs were treated with peptide concentrations of 5, 10, 15, 20, 30, and 50 μM, we measured the leakage of vesicles by measuring the dequenching of ANTS released into the medium. Changes in the fluorescence of 2 mL of ANTS/DPX LUV samples were monitored setting an excitation fluorescence at 385 nm (slit width, 5 nm) and a fluorescence emission at 512 nm (slit width, 5 nm) for 10 min after the addition of the peptide. The percentage of liposomes leakage was calculated as %leakage = (*F*_i_ – *F*_0_)/(*F*_t_ – *F*_0_), where *F*_0_ represents the fluorescence of intact LUVs before the addition of peptide, and F_i_ and F_t_ are the intensities of the fluorescence achieved after peptide and Triton-X treatment, respectively.

### Structural Analyses

#### CD Spectroscopy

CD spectra of peptides **9**, **12**, **17**, and **24**–**26** were recorded using quartz cells of 0.1 cm path length with a JASCO J-710 CD spectropolarimeter at 25 °C. All spectra were measured in the 260–190 nm spectral range, 1 nm bandwidth, 4 accumulations, and 100 nm/min scanning speed.^60^ Each peptide was dissolved in water to prepare a 1 mM peptide stock solution. The CD spectra were performed in water, in SDS (20 mM) and in DPC (20 mM) using a peptide concentration of 50 μM. The secondary structure content of the peptides was predicted using the online server for protein secondary structure analyses DichroWeb.^61^ Input and output units and the wavelength step were θ (mdeg) and 1.0 nm, respectively. The algorithm used was CONTIN-LL,^83^ and reference database was set-7. When we have NRMSD > 0.100, we used the CDSSTR method.^83^

CD spectra using SUVs were recorded only for linear peptide **9** and cyclic peptides **12** and **17**. SUVs were prepared as reported: lipids (Gram-positive, DOPG/CL, 58/42 ratio in moles; Gram-negative DOPG/DOPE/CL, 63/23/12 ratio in moles) were dissolved in chloroform and an identical volume of peptide solution dissolved in TFE was added.^76^ Then, the samples were vortexed and lyophilized overnight. CD spectra were recorded at a peptide concentration of 8 μM and at a lipid final concentration of 100 μM. For the measurement, the SUVs with peptide were rehydrated with phosphate buffer 5 mM, pH = 7.4 for 1 h and sonicated for 30 min. CD spectra were recorded at 25 °C on a Jasco J-715 spectropolarimeter in a 1 cm quartz cell using three consecutive scans from 260 to 190 nm, 3 nm bandwidth, a time constant of 16 s, and a scan rate of 10 nm/min.

#### NMR Spectroscopy

The samples for NMR spectroscopy were prepared by dissolving the appropriate amount of peptide **12** in 0.18 mL of ^1^H_2_O, 0.02 mL of ^2^H_2_O to obtain a concentration of 1–2 mM peptides, and 180 mM SDS-d_25_/20 mM DPC-d_38_. The NMR experiments were performed at pH 5.0. NMR spectra were recorded at 298 K on a Bruker Avance NEO 700 MHz spectrometer equipped with a Z-gradient cryoprobe. All of the spectra were recorded at a temperature of 25 °C. The spectra were calibrated relative to TSP (0.00 ppm) as internal standard. One-dimensional (1D) NMR spectra were recorded in the Fourier mode with quadrature detection. Two-dimensional (2D) DQF-COSY,^84,85^ TOCSY,^86^ and NOESY spectra^87^ were recorded in the phase-sensitive mode using the method from States et al.^88^ Data block sizes were 2048 addresses in t2 and 512 equidistant t1 values. A mixing time of 80 ms was used for the TOCSY experiments. NOESY experiments were run with a mixing time of 100 ms. The water signal was suppressed by gradient echo.^89^ The 2D NMR spectra were processed using the NMRPipe package.^90^ Before Fourier transformation, the time domain data matrices were multiplied by shifted sin2 functions in both dimensions, and the free induction decay size was doubled in F1 and F2 by zero filling. The qualitative and quantitative analyses of DQF-COSY, TOCSY, and NOESY spectra were obtained using the interactive program package XEASY.^91^^3^J_HN_–H_α_ couplings were difficult to measure probably because of a combination of small coupling constants and broad lines. The temperature coefficients of the amide proton chemical shifts were calculated from 1D ^1^H NMR and 2D TOCSY experiments performed at different temperatures in the range 298–320 K by means of linear regression.

#### Structural Determinations

The NOE-based distance restraints were obtained from NOESY spectra collected with a mixing time of 100 ms. The NOE cross-peaks were integrated with the XEASY program and were converted into upper distance bounds using the CALIBA program incorporated into the program package CYANA.^92^ Only NOE-derived constraints were considered in the annealing procedures. An ensemble of 200 structures was generated with the simulated annealing of the program CYANA. Then, 20 structures were chosen, whose interproton distances best fitted NOE-derived distances, and refined through successive steps of restrained and unrestrained energy minimization calculations using the Discover algorithm (Accelrys, San Diego, CA) and the consistent valence force field.^93^ The minimization lowered the total energy of the structures; no residue was found in the disallowed region of the Ramachandran plot. The final structures were analyzed using the InsightII program (Accelrys, San Diego, CA). Molecular graphics images were realized using the UCSF Chimera package.^94^

### Proteolytic Stability Assay

Human serum from human male AB plasma was acquired by Sigma-Aldrich/Merck. The proteolytic stability of linear peptide **9** and lactam-bridged analogue **12** was determined by employing the analytical RP-UHPLC was performed on a Shimadzu Nexera, equipped with a Phenomenex Kinetex column (C18, 4.6 mm×150 mm, 5 mm) and H_2_O (0.1% TFA) and MeCN (0.1% TFA) as eluents, as elsewhere described.^56^ Peptides **9** and **12** were dissolved in sterile water to prepare stock solutions of 2 mM. The peptide was incubated in a 1.5 mL eppendorf tube with human serum at a final concentration of 0.2 mM, and the mixture was incubated at 37 ± 1 °C for different periods (0.25, 0.75, 1.5, 2, 3, 4, 6, 7, 8, and 12 h). After the incubation period, an aliquot of reaction solution was taken and added MeCN (double volume respect to the aliquot) for the precipitation of serum proteins present in human serum. The produced cloudy solution was cooled (4 °C) for 15 min and then centrifuged at 13 000 rpm for 10 min to remove the serum protein as pellet. Then, the supernatant was analyzed by RP-UHPLC using a linear elution gradient from 10 to 90% MeCN (0.1% TFA) in water (0.1% TFA) over 20 min. A flow rate of 1 mL/min was employed, and the absorbance of the eluting peaks was detected at 220 nm. The proteolytic stability assay was repeated in triplicate.

## Supporting Information Available

Synthetic paths to achieve peptides **12**, **17**, **25**, and **26**; analytical data of peptides **10**–**26**; NMR data of peptide **12**; NMR-based restraints used in the structure calculation; and HPLC chromatograms of peptides **10**–**26** (PDF)
Molecular formula strings (CSV)

## Accession Codes

PDB code for **9** is 7OS8; PDB code for **12** is 7OSD.

## Abbreviations

AMP: antimicrobial peptide
ANTS: 8-aminonaphtalene-1,3,6-trisulfonic acid, disodium salt
Az: azidolysine
COMU: morpholino-carbenium hexafluorophosphate
CuAAC: copper-catalyzed azide–alkyne cycloaddition
DPC: dodecylphosphocholine
DPX: *p*-xylene-bis-pyridinium bromide
HOAt: 1-hydroxyazabenzotriazole
Oxyma: ethyl cyano(hydroxyimino)acetate
Pra: propargylalanine
PyAOP: (7-azabenzotriazol-1-yloxy)tripyrrolidinophosphonium hexafluorophosphate
RP-HPLC: reverse-phase high-pressure liquid chromatography
S_5_: (*S*)-2-(4-pentenyl)alanine
ThT: Thioflavin T
TL: temporin L
US–SPPS: ultrasonic-assisted solid-phase peptide synthesis
