# Supplementary material for: First-in-Class Cyclic Temporin L Analogue: Design, Synthesis, and Antimicrobial Assessment
Source: J Med Chem. 2021 Jul 23;64(15):11675–94. doi: 10.1021/acs.jmedchem.1c01033 (PMC8389922; doi:10.1021/acs.jmedchem.1c01033)
Supplement: Supplementary file 1 — jm1c01033_si_001.pdf [file jm1c01033_si_001.pdf]

Supporting information

First-in-class cyclic Temporin L analogue: design, synthesis and antimicrobial assessment

Rosa Bellavita,[1,§] Bruno Casciaro,[2,§] Salvatore Di Maro,[3] Diego Brancaccio,[1] Alfonso Carotenuto,[1] Annarita Falanga,[4] Floriana Cappiello,[5] Elisabetta Buommino,[1] Stefania Galdiero,[1] Ettore Novellino,[1] Tom N. Grossmann,[6] Maria Luisa Mangoni,[5] Francesco Merlino[1,*] and Paolo Grieco[1]

[1]Department of Pharmacy, University of Naples “Federico II”, Naples, 80131, Italy; [2]Center for Life Nano- & Neuro-Science, Fondazione Istituto Italiano di Tecnologia (IIT), Rome, 00161, Italy; [3]DiSTABiF, University of Campania “Luigi Vanvitelli”, Caserta, 81100, Italy; [4]Department of Agricultural Sciences, University of Naples “Federico II”, Portici, 80055, Italy; [5]Department of Biochemical Sciences, Laboratory affiliated to Istituto Pasteur Italia-Fondazione Cenci Bolognetti, Sapienza University of Rome, Rome, 00185, Italy; [6]Department of Chemistry & Pharmaceutical Sciences, VU University Amsterdam, Amsterdam, 1081 HZ, The Netherlands.

[§] These authors contributed equally

[*]corresponding author’s email: francesco.merlino@unina.it

Table of contents

1. Synthetic paths for the achievement of key peptides **12**, **17**, **25** and **26** (Schemes S1-4)	S2
2. Analytical data of stapled peptides **10-26** (Table S1)	S5
3. NMR data of peptide **12** (Table S2)	S6
4. NMR-based restraints used in the structure calculation (Table S3)	S7
5. HPLC chromatograms of stapled peptides **10-26** (Figures S1-17)	S10

1. Synthetic paths for the achievement of key peptides **12**, **17**, **25** and **26**.

Path A:

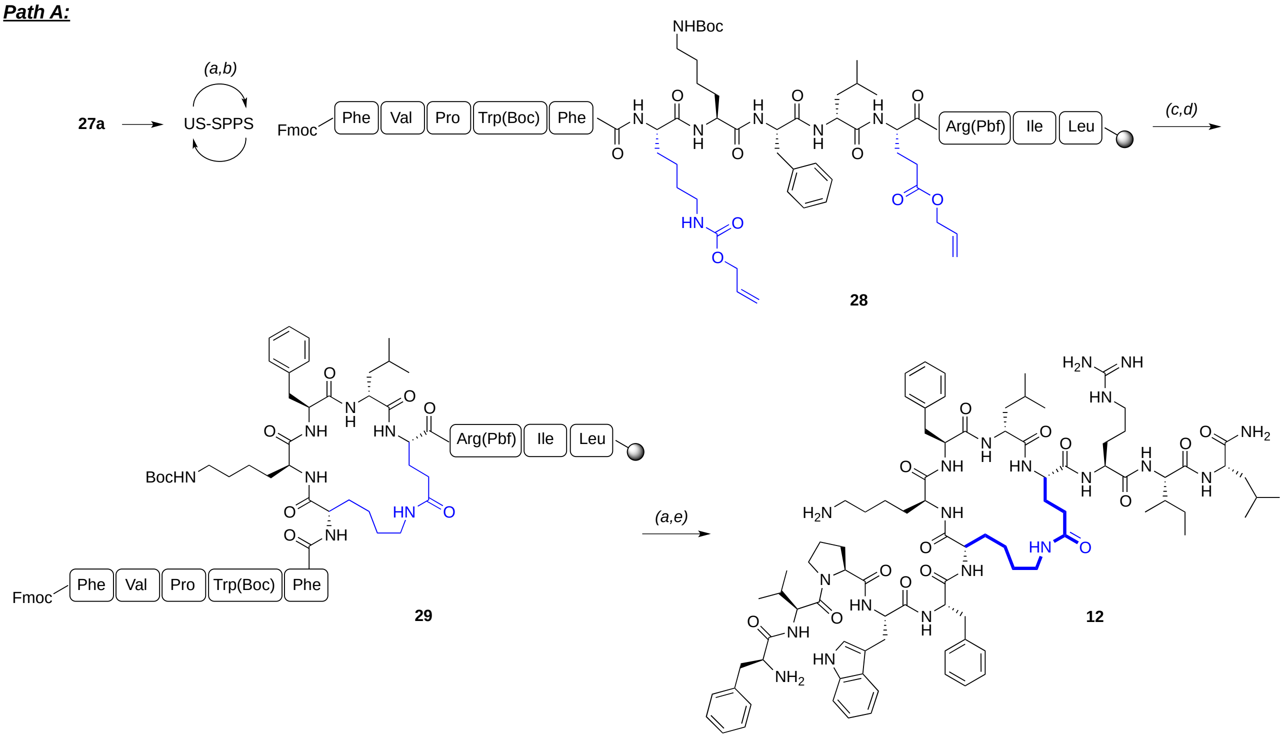

Scheme S1. (a) US-SPPS - Fmoc-deprotection: 20% piperidine in DMF, 0.5 + 1 min; (b) US-SPPS coupling: Fmoc-AA (2 equiv), COMU (2 equiv), Oxyma (2 equiv), DIEA (4 equiv), 5 min; (c) $Pd(PPh_3)_4$ (0.15 equiv), 1,3-dimethylbarbituric acid (3 equiv), DCM/DMF (3:2, *v/v*), 2 × 1 h; (d) PyAOP (2 equiv), HOAt (2 equiv), DIEA (4 equiv), DCM/DMF (1:1, *v/v*), 16 h; (e) TFA/TIS/H_2O (95:2.5:2.5, *v/v/v*), 3 h.

Path B:

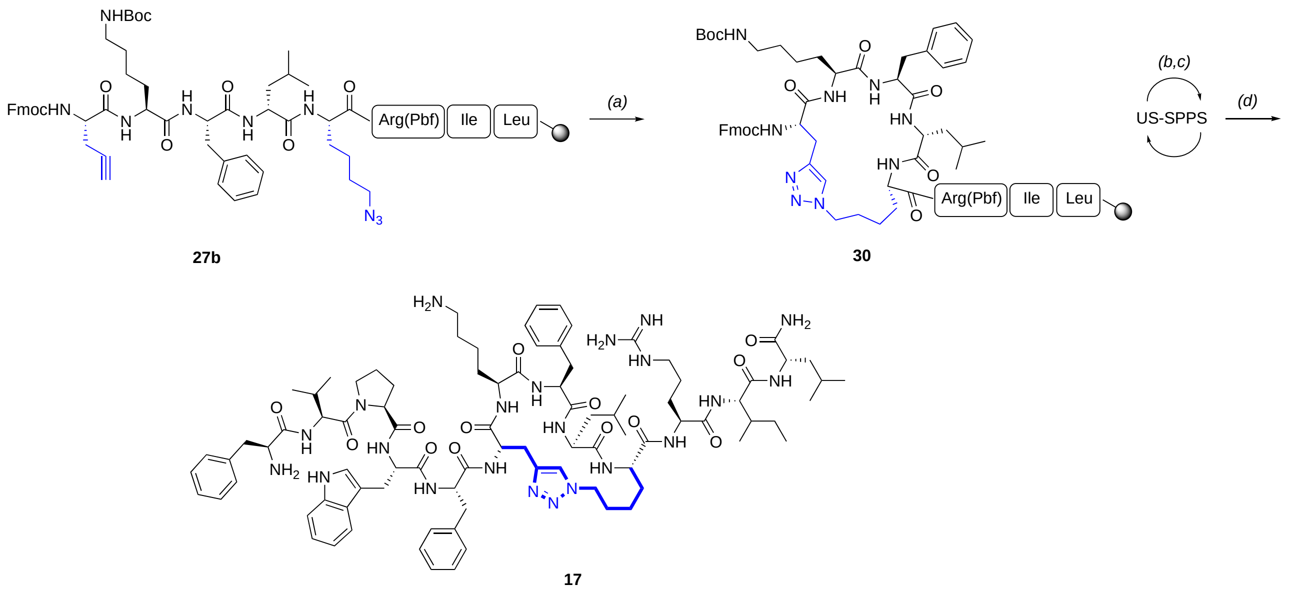

Scheme S2. (a) CuI (1 equiv), ascorbic acid (3 equiv), 2,4,6-collidine (5 equiv), DIEA (10 equiv), DMF, 3 × 1 h; (b) US-SPPS Fmoc-deprotection: 20% piperidine in DMF, 0.5 + 1 min; (c) US-SPPS coupling: Fmoc-AA (2 equiv), COMU (2 equiv), Oxyma (2 equiv), DIEA (4 equiv), 5 min; (d) TFA/TIS/H_2O (95:2.5:2.5, *v/v/v*), 3 h.

Path C:

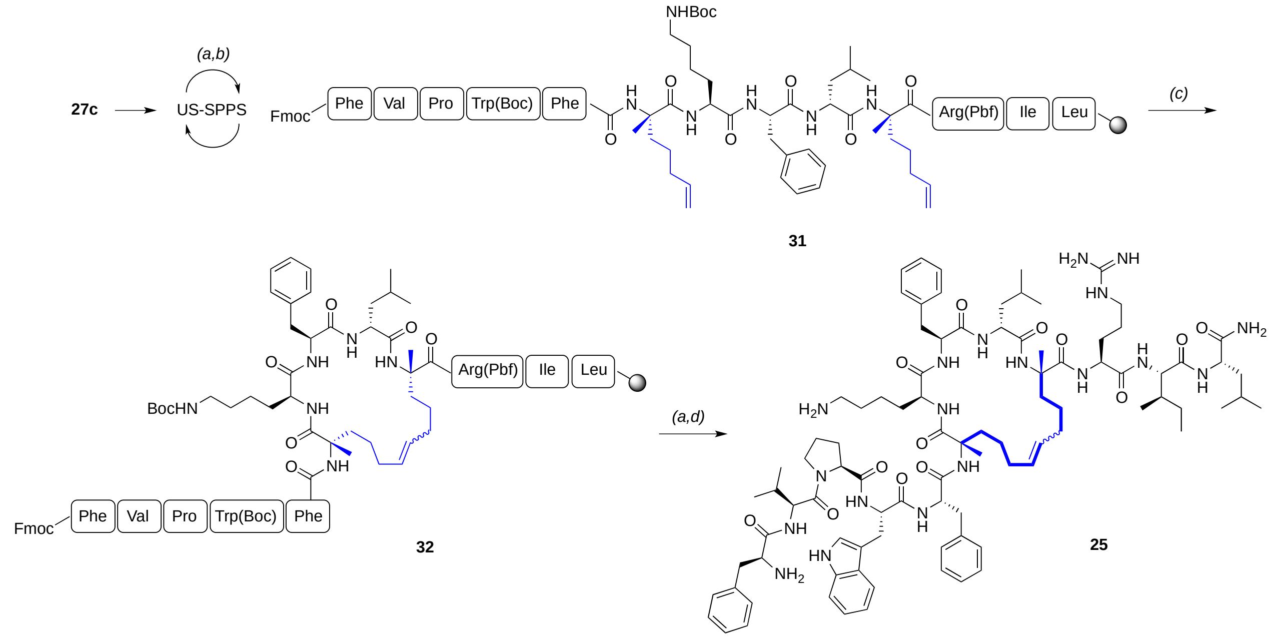

Scheme S3. (a) US-SPPS Fmoc-deprotection: 20% piperidine in DMF, 0.5 + 1 min; (b) US-SPPS coupling: Fmoc-AA (2 equiv), COMU (2 equiv), Oxyma (2 equiv), DIEA (4 equiv), 5 min; (c) 6 mM solution (3 mL) of the Grubbs' 1st generation catalyst in DCE (20 mol% relative to the resin substitution), 3 × 2 h, under nitrogen; (d) TFA/TIS/H_2O (95:2.5:2.5, *v/v/v*), 3 h.

Path D:

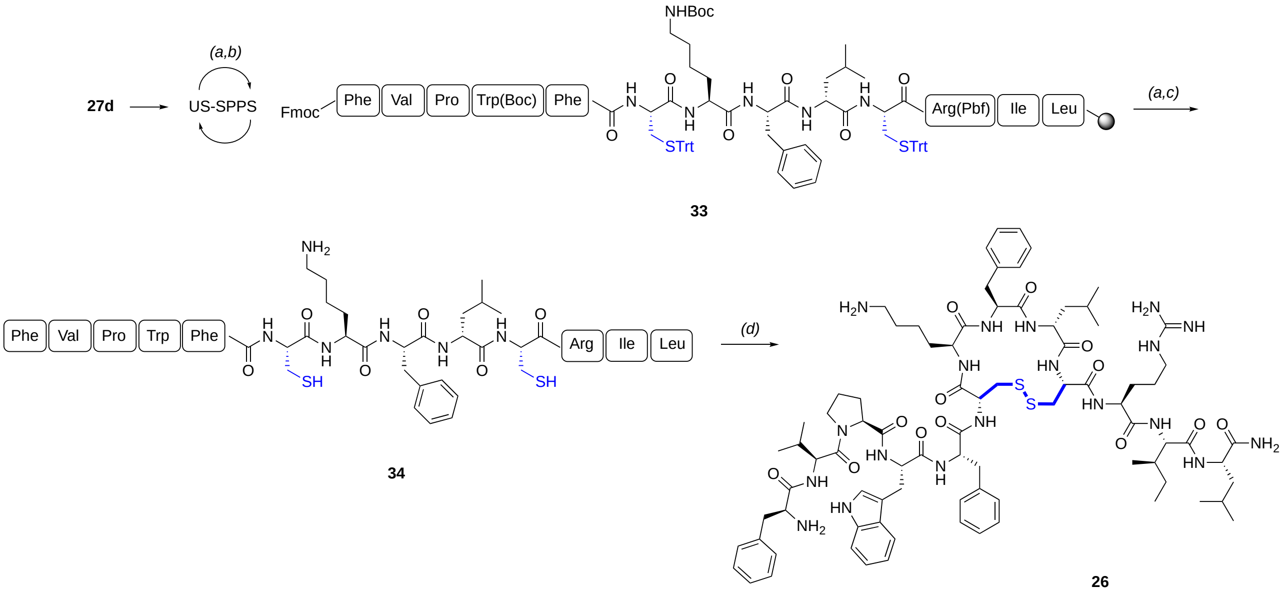

Scheme S4. (a) US-SPPS Fmoc-deprotection: 20% piperidine in DMF, 0.5 + 1 min; (b) US-SPPS coupling: Fmoc-AA (2 equiv), COMU (2 equiv), Oxyma (2 equiv), DIEA (4 equiv), 5 min; (c) TFA/TIS/H_2O (95:2.5:2.5, *v/v/v*), 3 h; (d) NCS (1 equiv), H_2O for [peptide]= 0.5 mM, 15 min.

Table S1. Analytical data for peptides **10-26**.

ID	t_R (min)	Molecular Formula	$[M+H]^+_{calcd}$	$[M+2H]^+/2_{obs}$
**10**	11.6	$C_{82}H_{117}N_{20}O_{15}^+$	1621.9002	811.9556
**11**	11.4	$C_{79}H_{119}N_{20}O_{15}^+$	1587.9158	794.4611
**12**	13.9	$C_{89}H_{131}N_{20}O_{14}^+$	1704.0148	853.0122
**13**	11.3	$C_{79}H_{118}N_{20}O_{15}^+$	1586.9080	794.9626
**14**	14.7	$C_{74}H_{119}N_{19}O_{15}^+$	1513.9128	757.9648
**15**	11.6	$C_{82}H_{115}N_{22}O_{14}^+$	1631.8958	816.4502
**16**	11.3	$C_{79}H_{117}N_{22}O_{14}^+$	1597.9114	799.9601
**17**	14.5	$C_{89}H_{129}N_{22}O_{13}^+$	1714.0104	858.0162
**18**	13.2	$C_{79}H_{117}N_{22}O_{14}^+$	1597.9114	799.9601
**19**	11.7	$C_{74}H_{118}N_{21}O_{14}^+$	1524.9162	761.9532
**20**	12.1	$C_{85}H_{122}N_{20}O_{14}^+$	1646.9444	824.9761
**21**	11.4	$C_{79}H_{119}N_{20}O_{15}^+$	1587.9158	794.4587
**22**	11.6	$C_{85}H_{121}N_{22}O_{13}^+$	1657.9478	829.9784
**23**	11.2	$C_{79}H_{117}N_{22}O_{14}^+$	1597.9114	799.4593
**24**	13.9	$C_{89}H_{131}N_{20}O_{14}^+$	1704.0148	853.0121
**25**	14.9	$C_{92}H_{136}N_{19}O_{13}^+$	1715.0560	858.0627
**26**	14.2	$C_{84}H_{122}N_{19}O_{13}S_2^+$	1668.8905	835.8973

Table S2. ^{1}H NMR resonance assignments[a] of peptide **12** in SDS/DPC 9:1 solution

residue	NH (-Δδ/ΔT)[b]	$C^{\alpha}H$	$C^{\beta}H$	Others
Phe 1		4.43	3.23, 3.17	7.73(δ); 7.27 (ε)
Val 2	7.74 (3.7)	4.19	1.79	0.69, 0.63(γ)
Pro 3		4.39	2.26	2.01(γ); 3.70(δ)
Trp 4	7.56 (2.9)	4.29	3.26. 3.19	7.36(δ); 10.00, 7.21(ε); 7.29, 6.81(ζ); 6.88(η)
Phe 5	7.84 (nd)	4.19	2.89, 2.62	7.08(δ); 7.17(ε)
Lys* 6	7.84 (7.5)	3.85	1.56, 1.36	1.19(γ); 1.46(δ); 2.65(ε)
Lys 7	7.81 (2.9)	3.97	1.85	1.48(γ); 1.66(δ); 2.94(ε); 7.38(ζ)
Phe 8	8.19 (5.2)	4.24	3.07, 2.97	7.11(δ); 7.16(ε)
DLeu 9	8.63 (5.0)	3.69	1.78	1.387(γ); 0.70, 0.64(δ)
Glu*10	7,94 (2.9)	3.82	1.97	2.31, 2.20(γ)
Arg 11	7.96 (2.2)	4.07	1.97	1.70, 1.59(γ); 3.25, 3.13(δ); 7.26(ε)
Ile 12	7.51 (3.1)	3.91	1.72	1.04, 1.00, 0.84 (γ); 0.76(δ)
Leu 13	8.04 (6.0)	4.26	1.53	1.68(γ); 0.85(δ)

[a] Obtained at 25 °C, pH= 5.5, with TSP (δ 0.00 ppm) as reference shift. Chemical shifts are accurate to ±0.02 ppm. -Δδ/ΔT = temperature coefficients (ppb/K); nd: not determined. $CONH_2$ (C-terminal amide protons) 7.00, 6.86. *Cyclic residues.

Table S3. NOE derived upper limit constraints used for peptide **12** structure calculation.

1	PHE	HA	1	PHE	HB2	3.02
1	PHE	HA	1	PHE	HB3	3.02
1	PHE	HA	1	PHE	QD	5.64
1	PHE	HA	2	VAL	HN	2.74
1	PHE	HB2	2	VAL	HN	3.55
1	PHE	HB3	2	VAL	HN	3.55
1	PHE	QD	2	VAL	HN	6.88
1	PHE	QD	2	VAL	HA	7.22
1	PHE	QD	2	VAL	QG1	8.40
1	PHE	QD	2	VAL	QG2	8.40
1	PHE	QD	2	VAL	QQG	6.93
1	PHE	QE	2	VAL	HA	7.62
1	PHE	QE	2	VAL	QG1	8.65
1	PHE	QE	2	VAL	QG2	8.65
2	VAL	HN	2	VAL	HB	3.05
2	VAL	HN	2	VAL	QQG	4.15
2	VAL	HN	3	PRO	QD	6.16
2	VAL	HA	2	VAL	QG1	3.83
2	VAL	HA	2	VAL	QG2	3.83
2	VAL	HA	3	PRO	QD	3.87
2	VAL	HB	3	PRO	QD	5.45
2	VAL	HB	4	TRP	HD1	5.07
2	VAL	HB	4	TRP	HE1	4.85
2	VAL	QG1	4	TRP	HE1	5.78
2	VAL	QG2	4	TRP	HE1	5.78
2	VAL	QQG	4	TRP	HD1	5.35
2	VAL	QQG	4	TRP	HE1	4.95
3	PRO	HA	7	LYS	HN	3.92
3	PRO	QD	4	TRP	HD1	6.38
4	TRP	HN	4	TRP	HB2	3.42
4	TRP	HN	4	TRP	HB3	3.42
4	TRP	HN	4	TRP	QB	2.96
4	TRP	HN	4	TRP	HD1	3.36
4	TRP	HN	5	PHE	HN	4.17
4	TRP	HA	4	TRP	HB2	2.80
4	TRP	HA	4	TRP	HB3	2.80
4	TRP	HA	4	TRP	HD1	3.73
4	TRP	HA	4	TRP	HE3	3.89
4	TRP	HA	7	LYS	QB	4.49
4	TRP	HB2	4	TRP	HD1	3.61
4	TRP	HB3	4	TRP	HD1	3.61
4	TRP	QB	4	TRP	HD1	3.07
4	TRP	QB	4	TRP	HE3	3.38
5	PHE	HN	5	PHE	HB2	3.52
5	PHE	HN	5	PHE	HB3	3.52
5	PHE	HN	5	PHE	QB	3.01
5	PHE	HA	5	PHE	QD	6.85
5	PHE	HA	8	PHE	HN	5.50
6	LYSS	HN	8	PHE	HN	4.23
6	LYSS	HA	7	LYS	HN	3.39
6	LYSS	HA	9	DLEU	HN	3.76
6	LYSS	QB	6	LYSS	QE	4.06
7	LYS	HN	7	LYS	HA	2.80

7	LYS	HN	7	LYS	QB	3.62
7	LYS	HN	7	LYS	QG	5.20
7	LYS	HN	7	LYS	QD	5.17
7	LYS	HN	8	PHE	HN	3.08
7	LYS	HA	10	GLUS	HN	3.61
7	LYS	HA	10	GLUS	QG	4.03
7	LYS	QB	7	LYS	QE	7.25
7	LYS	QB	7	LYS	QZ	7.19
7	LYS	QG	7	LYS	QZ	7.19
8	PHE	HN	8	PHE	HB2	3.17
8	PHE	HN	8	PHE	HB3	3.17
8	PHE	HN	8	PHE	QD	7.62
8	PHE	HN	9	DLEU	HN	3.64
8	PHE	HA	8	PHE	HB2	2.93
8	PHE	HA	8	PHE	HB3	2.93
8	PHE	HA	8	PHE	QB	2.55
8	PHE	HA	8	PHE	QD	5.45
8	PHE	HA	8	PHE	QE	6.35
8	PHE	HA	11	ARG+	HN	3.76
8	PHE	HA	11	ARG+	QB	4.33
8	PHE	HA	12	ILE	HN	4.32
8	PHE	QB	9	DLEU	HN	3.40
8	PHE	QD	9	DLEU	HN	7.62
9	DLEU	HN	9	DLEU	QQD	5.35
9	DLEU	HN	10	GLUS	HN	3.36
9	DLEU	HA	9	DLEU	QD1	4.67
9	DLEU	HA	9	DLEU	QD2	4.67
9	DLEU	HA	9	DLEU	QQD	3.62
9	DLEU	HA	10	GLUS	HN	3.42
10	GLUS	HN	10	GLUS	QB	4.14
10	GLUS	HN	10	GLUS	HG2	3.33
10	GLUS	HN	10	GLUS	HG3	3.33
10	GLUS	HN	10	GLUS	QG	2.82
10	GLUS	HA	10	GLUS	HG2	3.79
10	GLUS	HA	10	GLUS	HG3	3.79
10	GLUS	HA	13	LEU	HN	3.61
10	GLUS	HA	14	CNH2	HN1	4.51
10	GLUS	HA	14	CNH2	HN2	4.85
11	ARG+	HN	11	ARG+	QB	4.11
11	ARG+	HN	12	ILE	HN	3.27
11	ARG+	HA	11	ARG+	HG2	3.76
11	ARG+	HA	11	ARG+	HG3	3.76
11	ARG+	HA	11	ARG+	QG	3.23
11	ARG+	HA	11	ARG+	QD	3.71
11	ARG+	HA	12	ILE	HN	3.64
11	ARG+	HA	14	CNH2	HN1	5.50
11	ARG+	QB	11	ARG+	HE	5.38
11	ARG+	QB	12	ILE	HN	4.24
11	ARG+	HG2	11	ARG+	HE	3.98
11	ARG+	HG3	11	ARG+	HE	3.98
11	ARG+	QG	11	ARG+	HE	3.37
11	ARG+	QG	12	ILE	HN	4.47
12	ILE	HN	12	ILE	HB	2.83
12	ILE	HN	12	ILE	QG2	4.67
12	ILE	HN	12	ILE	QG1	3.39
12	ILE	HN	12	ILE	QD1	5.54
12	ILE	HN	13	LEU	HN	2.74

12	ILE	HN	13	LEU	QQD	7.63
12	ILE	HN	14	CNH2	HN1	5.50
12	ILE	HA	12	ILE	QG2	3.68
12	ILE	HA	12	ILE	QG1	3.31
12	ILE	HA	12	ILE	QD1	4.08
12	ILE	HA	13	LEU	HN	3.36
12	ILE	HB	13	LEU	HN	2.90
12	ILE	QG2	12	ILE	QG1	3.30
12	ILE	QG2	13	LEU	HN	4.76
13	LEU	HN	13	LEU	HA	2.86
13	LEU	HN	13	LEU	QB	4.18
13	LEU	HN	13	LEU	HG	2.43
13	LEU	HN	13	LEU	QQD	6.02
13	LEU	HN	14	CNH2	HN1	3.30
13	LEU	HN	14	CNH2	HN2	3.92
13	LEU	HA	13	LEU	HG	3.33
13	LEU	HA	13	LEU	QQD	4.59
13	LEU	QB	14	CNH2	HN1	6.38
13	LEU	HG	14	CNH2	HN1	4.17
13	LEU	HG	14	CNH2	HN2	4.72
13	LEU	QQD	14	CNH2	HN1	7.63
13	LEU	QQD	14	CNH2	HN2	7.63

5. HPLC chromatograms of peptides **10-26:**

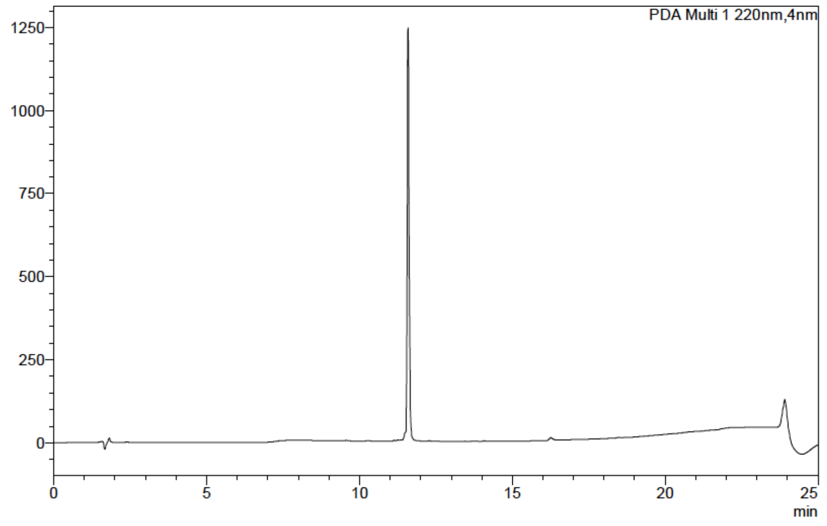

Figure S1. Chromatogram of **10** obtained by an analytical HPLC (Shimadzu UFLC SPD-M20A/DGU-20A3R/LC-20AD) equipped with a Phenomenex Kinetex C18 column (150 mm × 4.6 mm, 5 µm, 100 Å), and monitored by UV detection at 220 nm. t_R: 11.6 min [linear gradient 10-90% MeCN (0.1% TFA) in H_2O (0.1% TFA) over 20 min, flow rate of 1 mL/min].

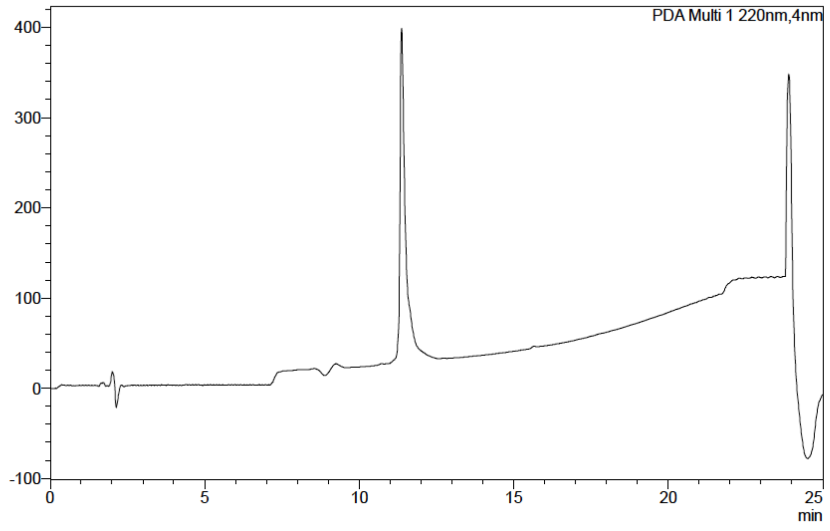

Figure S2. Chromatogram of **11** obtained by an analytical HPLC (Shimadzu UFLC SPD-M20A/DGU-20A3R/LC-20AD) equipped with a Phenomenex Kinetex C18 column (150 mm × 4.6 mm, 5 µm, 100 Å), and monitored by UV detection at 220 nm. t_R: 11.4 min [linear gradient 10-90% MeCN (0.1% TFA) in H_2O (0.1% TFA) over 20 min, flow rate of 1 mL/min].

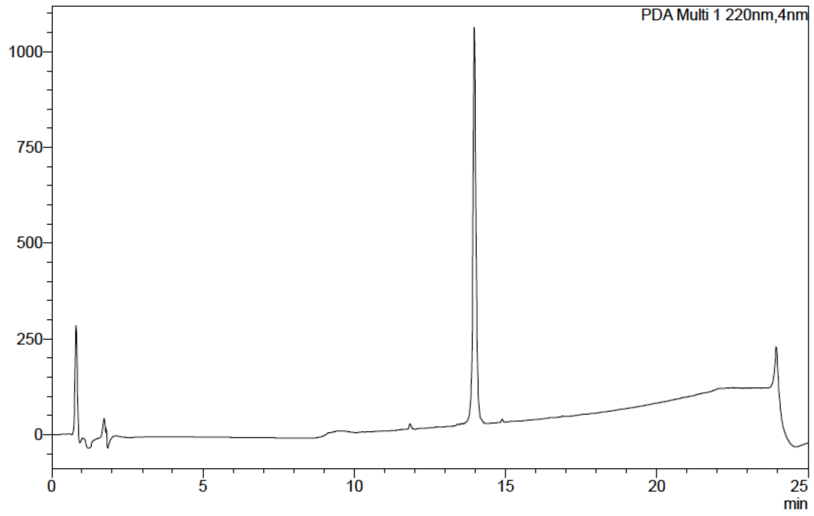

Figure S3. Chromatogram of **12** obtained by an analytical HPLC (Shimadzu UFLC SPD-M20A/DGU-20A3R/LC-20AD) equipped with a Phenomenex Kinetex C18 column (150 mm × 4.6 mm, 5 μm, 100 Å), and monitored by UV detection at 220 nm. t_R: 13.9 min [linear gradient 10-90% MeCN (0.1% TFA) in H_2O (0.1% TFA) over 20 min, flow rate of 1 mL/min].

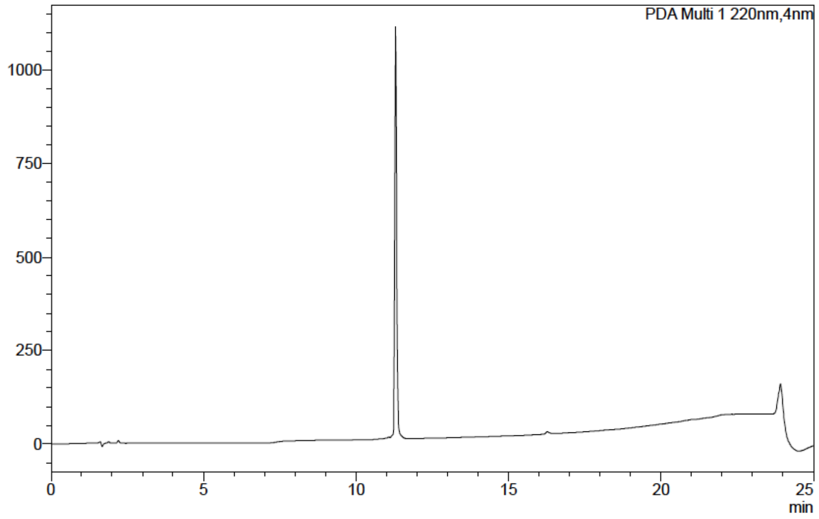

Figure S4. Chromatogram of **13** obtained by an analytical HPLC (Shimadzu UFLC SPD-M20A/DGU-20A3R/LC-20AD) equipped with a Phenomenex Kinetex C18 column (150 mm × 4.6 mm, 5 μm, 100 Å), and monitored by UV detection at 220 nm. t_R: 11.3 min [linear gradient 10-90% MeCN (0.1% TFA) in H_2O (0.1% TFA) over 20 min, flow rate of 1 mL/min].

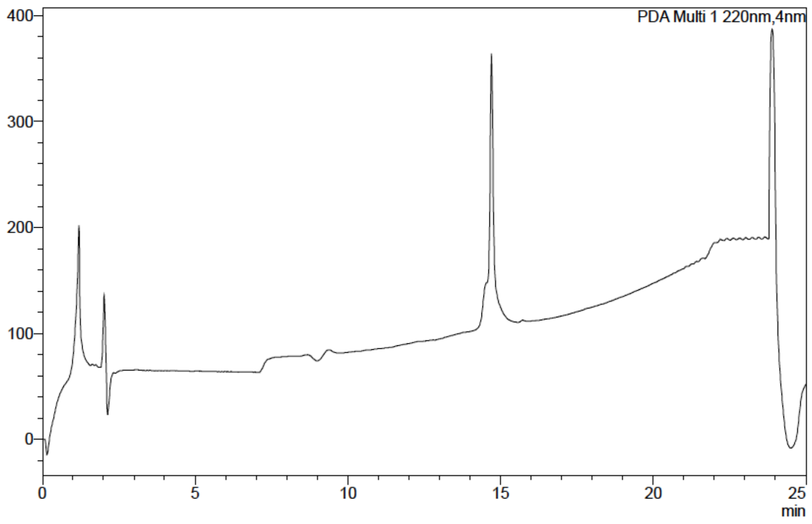

Figure S5. Chromatogram of **14** obtained by an analytical HPLC (Shimadzu UFLC SPD-M20A/DGU-20A3R/LC-20AD) equipped with a Phenomenex Kinetex C18 column (150 mm × 4.6 mm, 5 µm, 100 Å), and monitored by UV detection at 220 nm. t_R: 14.7 min [linear gradient 10-90% MeCN (0.1% TFA) in H_2O (0.1% TFA) over 20 min, flow rate of 1 mL/min].

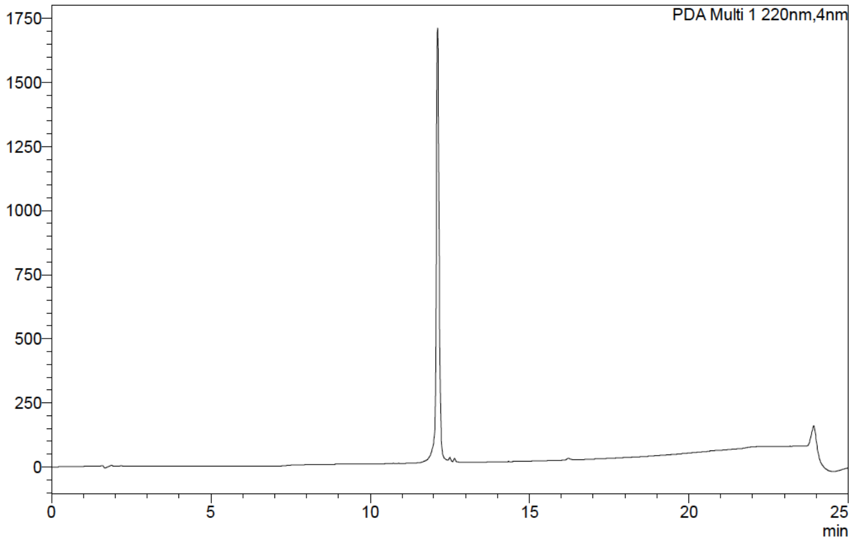

Figure S6. Chromatogram of **15** obtained by an analytical HPLC (Shimadzu UFLC SPD-M20A/DGU-20A3R/LC-20AD) equipped with a Phenomenex Kinetex C18 column (150 mm × 4.6 mm, 5 µm, 100 Å), and monitored by UV detection at 220 nm. t_R: 11.6 min [linear gradient 10-90% MeCN (0.1% TFA) in H_2O (0.1% TFA) over 20 min, flow rate of 1 mL/min].

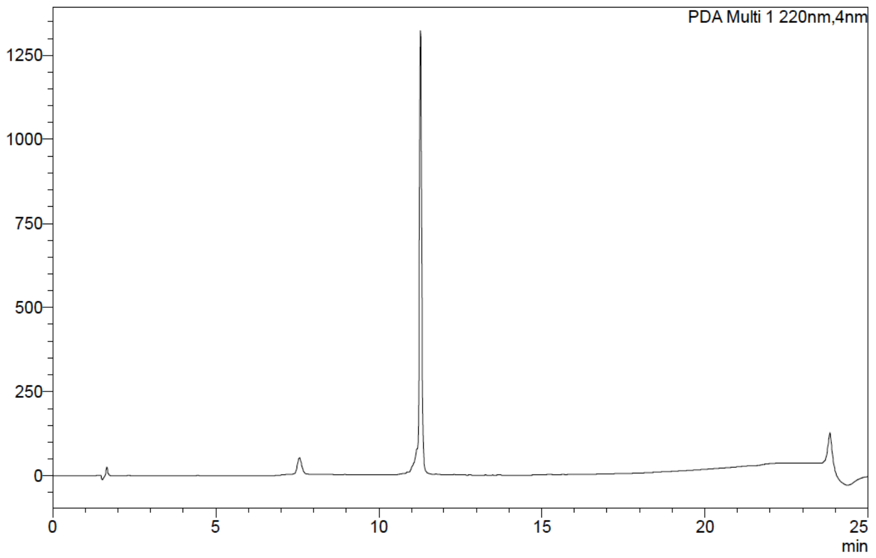

Figure S7. Chromatogram of **16** obtained by an analytical HPLC (Shimadzu UFLC SPD-M20A/DGU-20A3R/LC-20AD) equipped with a Phenomenex Kinetex C18 column (150 mm × 4.6 mm, 5 μm, 100 Å), and monitored by UV detection at 220 nm. t_R:11.3 min [linear gradient 10-90% MeCN (0.1% TFA) in H_2O (0.1% TFA) over 20 min, flow rate of 1 mL/min].

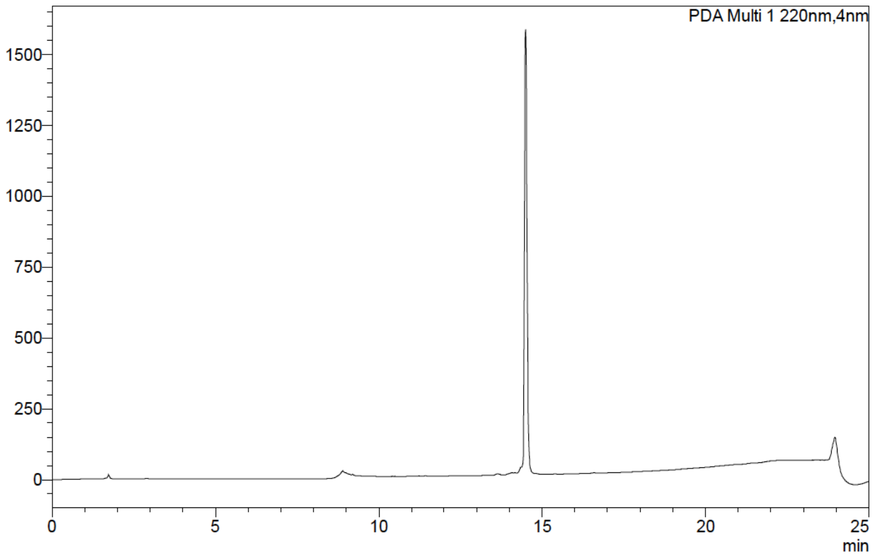

Figure S8. Chromatogram of **17** obtained by an analytical HPLC (Shimadzu UFLC SPD-M20A/DGU-20A3R/LC-20AD) equipped with a Phenomenex Kinetex C18 column (150 mm × 4.6 mm, 5 μm, 100 Å), and monitored by UV detection at 220 nm. t_R: 14.5 min [linear gradient 10-90% MeCN (0.1% TFA) in H_2O (0.1% TFA) over 20 min, flow rate of 1 mL/min].

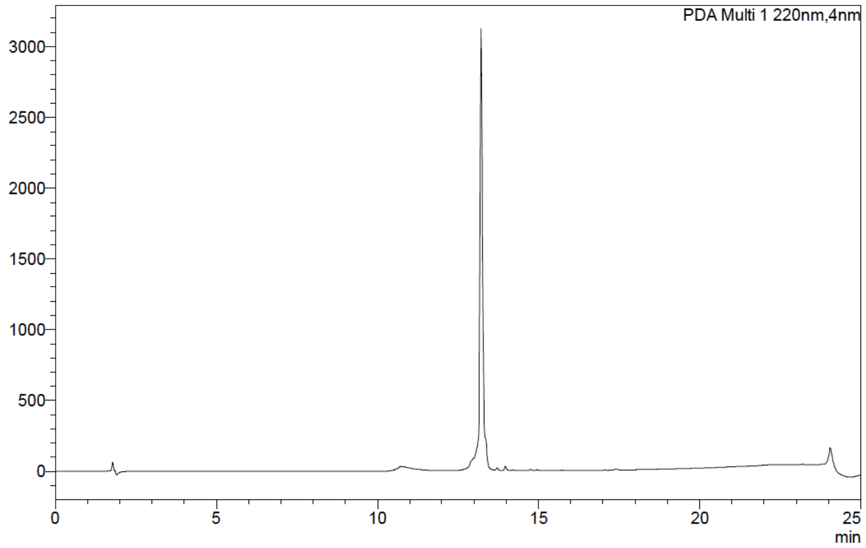

Figure S9. Chromatogram of **18** obtained by an analytical HPLC (Shimadzu UFLC SPD-M20A/DGU-20A3R/LC-20AD) equipped with a Phenomenex Kinetex C18 column (150 mm × 4.6 mm, 5 μm, 100 Å), and monitored by UV detection at 220 nm. t_R: 13.2 min [linear gradient 10-90% MeCN (0.1% TFA) in H_2O (0.1% TFA) over 20 min, flow rate of 1 mL/min].

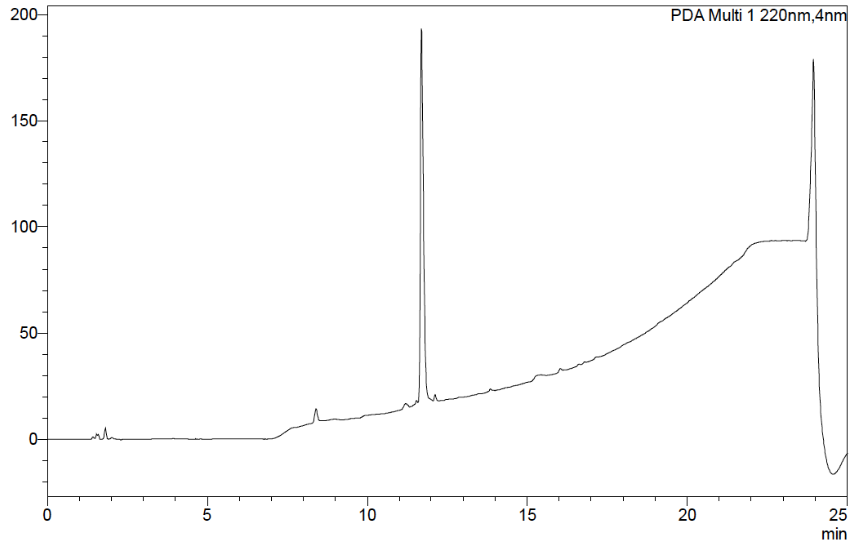

Figure S10. Chromatogram of **19** obtained by an analytical HPLC (Shimadzu UFLC SPD-M20A/DGU-20A3R/LC-20AD) equipped with a Phenomenex Kinetex C18 column (150 mm × 4.6 mm, 5 μm, 100 Å), and monitored by UV detection at 220 nm. t_R: 11.7 min [linear gradient 10-90% MeCN (0.1% TFA) in H_2O (0.1% TFA) over 20 min, flow rate of 1 mL/min].

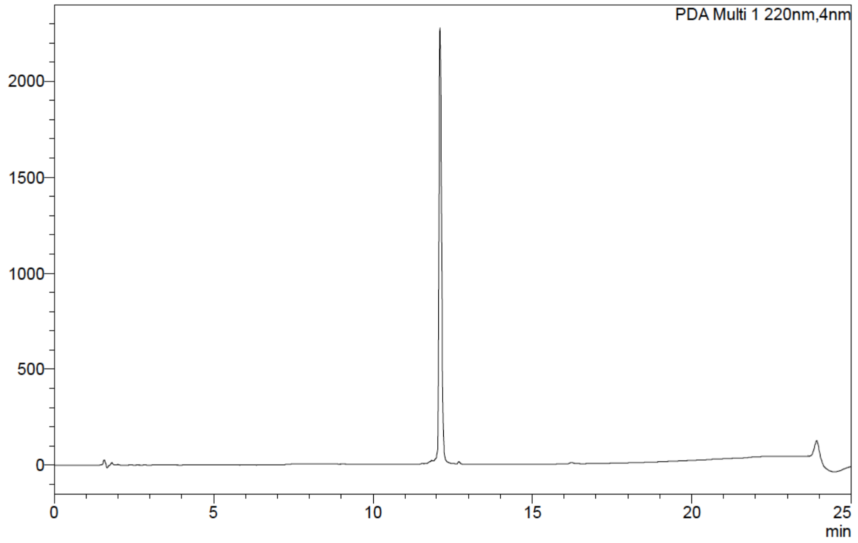

Figure S11. Chromatogram of **20** obtained by an analytical HPLC (Shimadzu UFLC SPD-M20A/DGU-20A3R/LC-20AD) equipped with a Phenomenex Kinetex C18 column (150 mm × 4.6 mm, 5 μm, 100 Å), and monitored by UV detection at 220 nm. t_R: 12.1 [linear gradient 10-90% MeCN (0.1% TFA) in H_2O (0.1% TFA) over 20 min, flow rate of 1 mL/min].

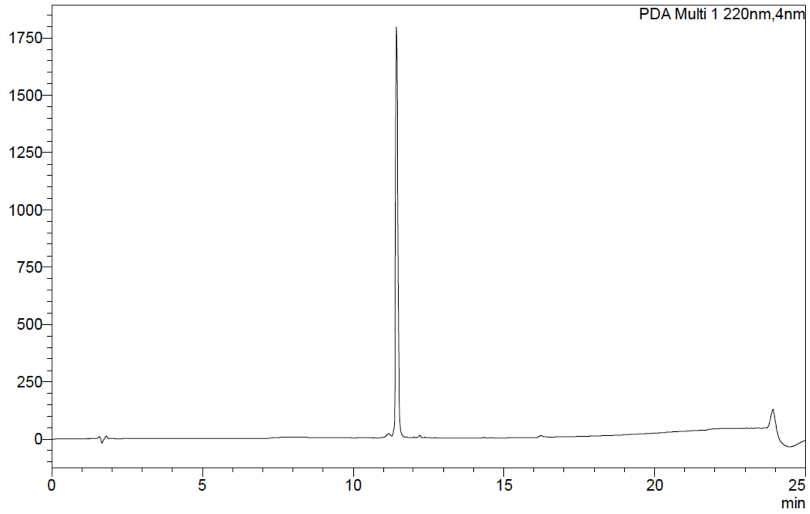

Figure S12. Chromatogram of **21** obtained by an analytical HPLC (Shimadzu UFLC SPD-M20A/DGU-20A3R/LC-20AD) equipped with a Phenomenex Kinetex C18 column (150 mm × 4.6 mm, 5 μm, 100 Å), and monitored by UV detection at 220 nm. t_R: 11.4 [linear gradient 10-90% MeCN (0.1% TFA) in H_2O (0.1% TFA) over 20 min, flow rate of 1 mL/min].

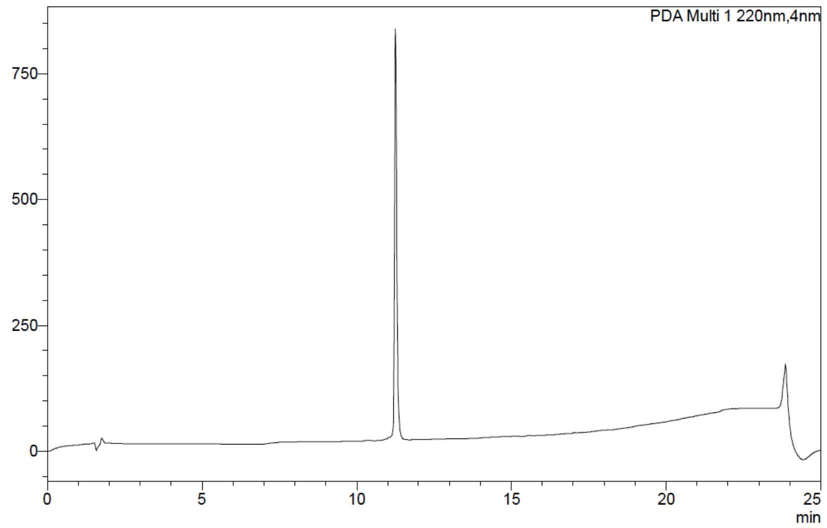

Figure S13. Chromatogram of **22** obtained by an analytical HPLC (Shimadzu UFLC SPD-M20A/DGU-20A3R/LC-20AD) equipped with a Phenomenex Kinetex C18 column (150 mm × 4.6 mm, 5 μm, 100 Å), and monitored by UV detection at 220 nm. t_R: 11.6 min [linear gradient 10-90% MeCN (0.1% TFA) in H_2O (0.1% TFA) over 20 min, flow rate of 1 mL/min].

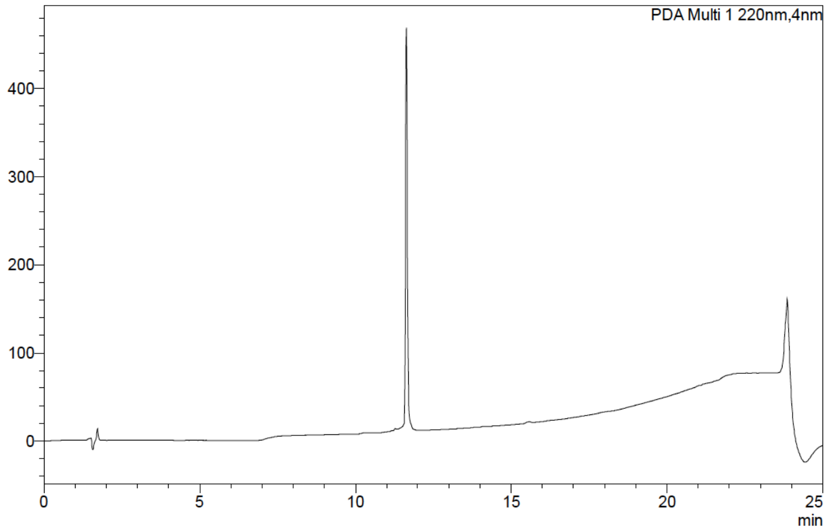

Figure S14. Chromatogram of **23** obtained by an analytical HPLC (Shimadzu UFLC SPD-M20A/DGU-20A3R/LC-20AD) equipped with a Phenomenex Kinetex C18 column (150 mm × 4.6 mm, 5 μm, 100 Å), and monitored by UV detection at 220 nm. t_R: 11.3 min [linear gradient 10-90% MeCN (0.1% TFA) in H_2O (0.1% TFA) over 20 min, flow rate of 1 mL/min].

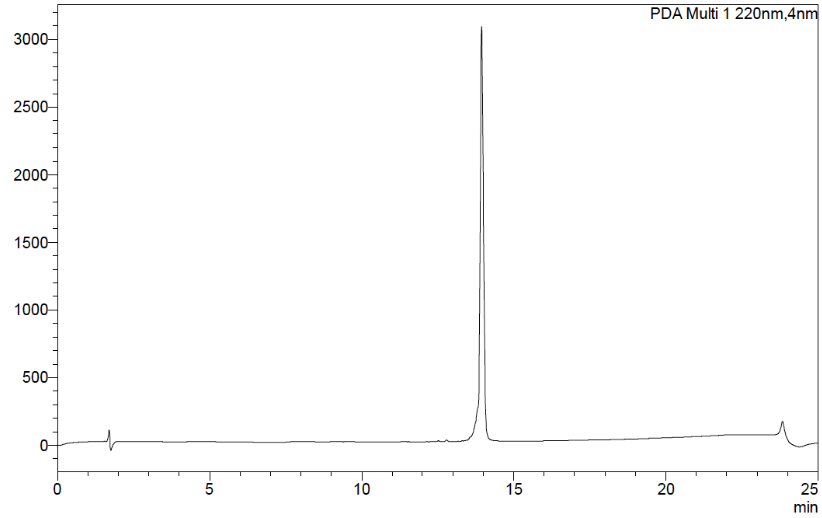

Figure S15. Chromatogram of **24** obtained by an analytical HPLC (Shimadzu UFLC SPD-M20A/DGU-20A3R/LC-20AD) equipped with a Phenomenex Kinetex C18 column (150 mm × 4.6 mm, 5 µm, 100 Å), and monitored by UV detection at 220 nm. t_R: 13.9 min [linear gradient 10-90% MeCN (0.1% TFA) in H_2O (0.1% TFA) over 20 min, flow rate of 1 mL/min].

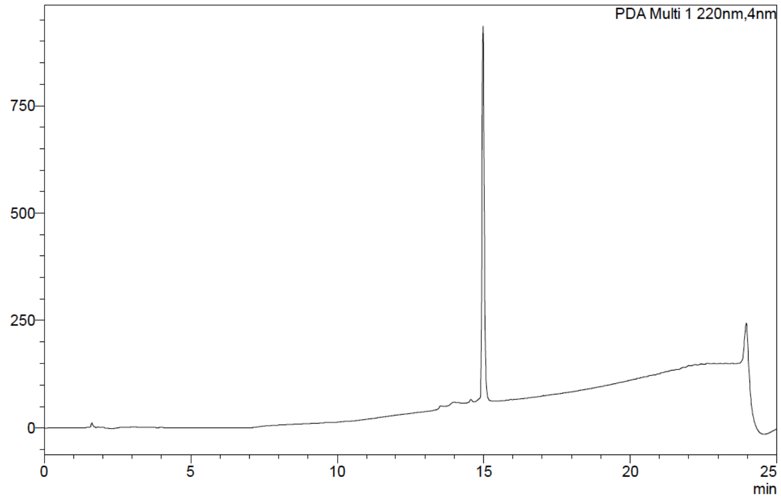

Figure S16. Chromatogram of **25** obtained by an analytical HPLC (Shimadzu UFLC SPD-M20A/DGU-20A3R/LC-20AD) equipped with a Phenomenex Kinetex C18 column (150 mm × 4.6 mm, 5 µm, 100 Å), and monitored by UV detection at 220 nm. t_R: 14.9 min [linear gradient 10-90% MeCN (0.1% TFA) in H_2O (0.1% TFA) over 20 min, flow rate of 1 mL/min].

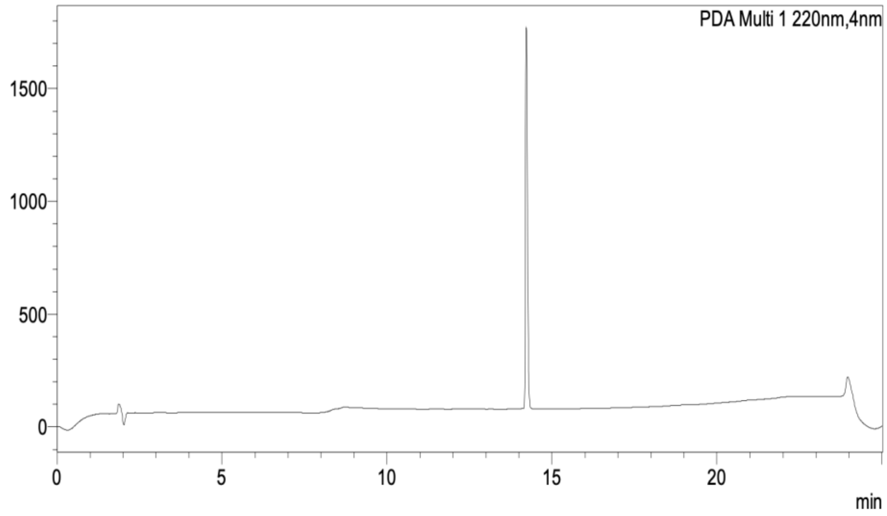

Figure S17. Chromatogram of **26** obtained by an analytical HPLC (Shimadzu UFLC SPD-M20A/DGU-20A3R/LC-20AD) equipped with a Phenomenex Kinetex C18 column (150 mm × 4.6 mm, 5 μm, 100 Å), and monitored by UV detection at 220 nm. t_R: 14.2 min [linear gradient 10-90% MeCN (0.1% TFA) in H_2O (0.1% TFA) over 20 min, flow rate of 1 mL/min].
